# First Example of Catalytic Synthesis of Cyclic S-Containing Di- and Triperoxides

**DOI:** 10.3390/molecules25081874

**Published:** 2020-04-18

**Authors:** Nataliya Makhmudiyarova, Irina Ishmukhametova, Lilya Dzhemileva, Vladimir D’yakonov, Askhat Ibragimov, Usein Dzhemilev

**Affiliations:** Institute of Petrochemistry and Catalysis, Russian Academy of Sciences, Ufa 450075, Russia; iuliania93@mail.ru (I.I.); dyakonovVA@gmail.com (V.D.) a.ibragimov@mail.ru (A.I.); dzhemilev@anrb.ru (U.D.)

**Keywords:** catalysis, lanthanide salts, hydrogen sulfide, thia-peroxides, cytotoxic activity

## Abstract

An efficient method for the synthesis of tetraoxathiaspiroalkanes, tetraoxathiocanes, and hexaoxathiadispiroalkanes was developed by reactions of pentaoxacanes, pentaoxaspiroalkanes, and heptaoxadispiroalkanes with hydrogen sulfide in the presence of a catalyst, Sm(NO_3_)_3_·6H_2_O. We found that the synthesized S-containing di- and triperoxides exhibit high cytotoxic activity against Jurkat, K562, U937, and HL60 tumor cultures, and fibroblasts.

## 1. Introduction

Cyclic peroxides occur widely in nature, and they often possess desired pharmacological properties. For example, an eight-membered cyclic azaperoxide moiety is included in the biologically active alkaloid compounds fumitremorgins [1,2,3,4,5,6,7], namely into the fumitremorgin A *Verruculogen* produced by fungi of species *Penicillium verruculosum* [8], *Aspergillus caespitosus* [9], *A. fumigatus* [10], *A. fischeri* [11], *Penicillium piscarium* [12], *Penicillium paxilli* [13], *Penicillium estinogenum* [14], *Penicillium simplicissimum, Penicillium piceum, Penicillium nigricans, Penicillium raistricki* [15], and *Neosartorya fischeri* [16]. Fumitremorgin and related compounds are active against various cancer cells [17]. Some of these natural compounds can arrest cancer cells in their cell cycle, and some can block ABC transporters and reverse resistance in chemotherapy. Assessment of structural–functional relationships enabled prediction of biological activity in peroxide compounds due to a presence of heteroatom in the α-position with regard to the peroxide group [18,19,20]. Previously, we synthesized azaperoxides and demonstrated the cytotoxic activity of these compounds [21,22,23,24]. In continuation of ongoing research on the synthesis of heteroatom-containing peroxides, we attempted to synthesize S-peroxides.

The data available on heteroatom-containing peroxides with high pharmacological activity [25,26,27,28,29,30,31,32,33,34,35,36,37,38,39] suggest that S-containing peroxides could be useful for the development of antimalarial and antibacterial agents. Those cyclic S-containing peroxides known from the literature are represented by thio-ozonides [40,41,42,43,44], obtained via photooxidation at a temperature of –78 °C. In most instances [40,41,42,43,44], these compounds are already unstable at 0 °C. There is no data available on stable S-containing cyclic diperoxides. This paper describes a catalytic method developed for the synthesis of cyclic thia-diperoxides with high yields and selectivity.

## 2. Results and Discussion

### 2.1. Chemistry

A classic example of the preparation of cyclic thioesters is recyclization of furan using hydrogen sulfide according to the Yuriev reaction at a temperature of 550 °C in the presence of Al_2_O_3_ [45]. Practically no information is available in the literature on the synthesis of cyclic thioesters at room temperature under the action of lanthanide catalysts. We developed a method for the preparation of thioperoxycarbocycles through the recyclization of pentaoxacanes and heptaoxadispiroalkanes with hydrogen sulfide under the action of lanthanide catalysts. We chose lanthanide catalysts due to their high activity in recyclization reactions involving primary amines, leading to cyclic *N*-containing di- and triperoxides [46,47,48,49,50].

**Scheme 1:**
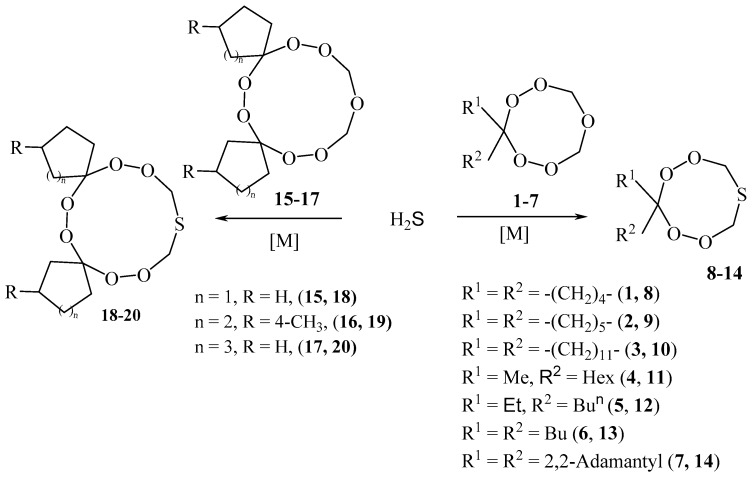
Synthesis of cyclic S-containing di- and triperoxides.

We assumed that cyclic thia-diperoxides may be synthesized by a reaction of pentaoxacanes with hydrogen sulfide in a similar manner to what we reported previously for the synthesis of cyclic aza-diperoxides via the reaction of pentaoxacanes with primary amines [46,47,50]. Preliminary experiments demonstrated that 7,8,10,12,13-pentaoxaspiro[5.7]tridecane [51] (**1**) reacts with H_2_S in the presence of the catalyst Sm(NO_3_)_3_·6H_2_O [46,47,48,49,50] for 6 h at room temperature in tetrahydrofuran (THF) solvent to produce 7,8,12,13-tetraoxa-10-thiaspiro[5.7]tridecane (**8**) in 98% yield. The reaction does not proceed in the absence of a catalyst (Scheme 1).

Subsequent experiments demonstrated that in certain conditions (5 mol % Sm(NO_3_)_3_^.^6H_2_O, 20 °C, 6 h), the yield of the target product **8** is dependent on the solvent and decreases in the following order: THF > CH_2_Cl_2_ > Et_2_O > C_6_H_12_ > EtOAc > C_2_H_5_OH (Table 1). To ascertain the dependency relationship between the nature of a central atom in the lanthanide catalyst and the yield of **8**, in the reaction presented here, we tested, along with the compound Sm(NO_3_)_3_·6H_2_O, a series of other lanthanide salts and complexes based on Ho, Tb, Dy, Nd, and La (Table 1). Use of the catalysts based on d- and f-elements, such as Co, Fe, Al, and Ni salts, results in decomposition of the peroxide group that enables the isolation of ketones and cyclic sulfides from the reaction mass. The reactions were conducted at ~20 °C in THF in the presence of the catalysts (5 mol %) specified earlier. Under the indicated conditions, selective formation of the 7,8,12,13-tetraoxa-10-thiaspiro[5.7]tridecane (**8**) was observed with yields of 58% to 84% (Table 1). In the determined conditions (5 mol % Sm(NO_3_)_3_·6H_2_O, THF, 20 °C, 6 h), the reaction of cyclocondensation of pentaoxaspiroalkanes (**2,3**) with H_2_S results in selective formation of tetraoxathiaspiroalkanes (**9,10**) in yields of 90% and 85%, respectively.

The reaction thus developed provides a convenient tool for preparation of various tetraoxathiocanes. By using the described procedure, the synthesis of 3,3-disubstituted tetraoxathiocanes was implemented via the catalytic reaction of pentaoxacanes with hydrogen sulfide. In reactions of 3,3-disubstituted pentaoxacanes **4**–**7** with H_2_S catalyzed by Sm(NO_3_)_3_·6H_2_O, 1,2,4,5,7-tetraoxathiocanes, **11**–**14** are selectively formed with yields of 80% to 89%.

**Scheme 2:**
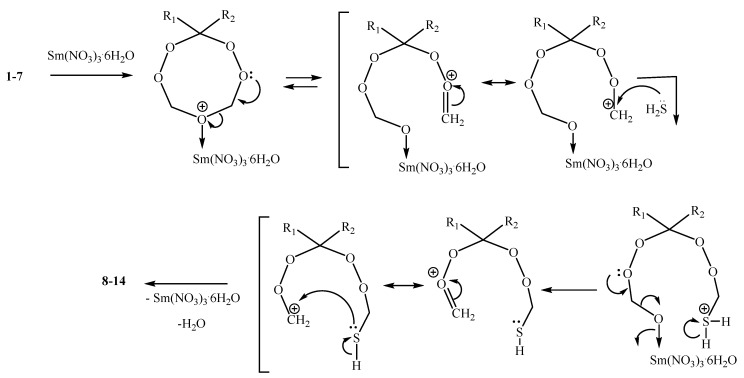
Formation of S-containing diperoxides (**8**–**14)**.

It can be assumed [52] that formation of tetraoxathiaspiroalkanes **8**–**14** occurs via a pentaoxacane ring opening affected by the catalyst [53,54]. Subsequent nucleophilic addition of H_2_S to the carbocation results in intramolecular cyclization, where the corresponding tetraoxathiaspiroalkanes **8**–**14** are obtained (Scheme 2). 

To expand the scope of applicability of the method for the synthesis of cyclic thio-peroxides developed here, we produced spiro-fused hexaoxathiocanes **18**–**20** by reaction of heptaoxacanes **15**–**17** [48] with hydrogen sulfide in THF (~20 °C, 6 h), catalyzed by Sm(NO_3_)_3_·6H_2_O (0.5 mol %). we observed that the size of the carbocycles in initial heptaoxadispiroalkanes **15**–**17** does not affect the yield of hexaoxathiocanes **18**–**20** (83–86%). 

The structures of cyclic S-containing peroxides 8–14 and 18–20 were confirmed by ^1^H and ^13^C NMR spectra of the synthesized compounds. The methylene fragment signals characteristic of these –S-CH_2_-O-O- systems are manifested in the regions of 4.81 to 5.31 ppm and 81.4 to 83.7 ppm in the spectra of ^1^H and ^13^C NMR, respectively. These signals reflect the process of cyclic interconversion in solution; therefore, we observed a set of signals with close chemical shifts for each of the individual compounds. The effect of the splitting of the NMR signals of the ring atoms is due to the presence of a multicomponent conformational equilibrium at room temperature, which can be assumed on the basis of published data on the identification of known heteroatom-containing peroxides, in particular azadi- and triperoxides [46,47,48,49,50]. The presence of one conformation was observed only in the case of 3-(adamantyl-2-yl)-1,2,4,5,7-tetraaoxatiocane (14), probably due to the rigidity of the structure of the spiroadamantane substituent.

### 2.2. Biological Evaluation

Cytotoxicity of azaperoxide-based compounds is well known [1,2,3,4,5,6,7,8,9,10,11,12,13,14,15,16,17,18], so we screened the representative compounds for their cytotoxicity activity against Jurkat, K562, U937, and HL60 fibroblasts cell lines. The results are summarized in Table 2.

The synthesized S-containing diperoxides **8**–**14** and triperoxides **18**–**20** exhibited a cytotoxic effect against a number of suspension tumor cell lines (Jurkat, K562, U937, and HL60) in the range of 2.24 to 65.81 µM and 79.17 to 195.87 µM for normal fibroblasts. The synthesized compounds had a rather high selectivity index (SI = IC_50_ fibroblasts/IC_50_ cancer cells) for Jurkat, HL60, and K562 tumor cells, ranging from 8 to 35, whereas for the U937 culture the selectivity index ranged from 3 to 7. The highest cytotoxic activity (2.24–11.79 µM) was exhibited by triperoxide **19**, synthesized based on 4-methylcyclohexane derivative **16**, as well as a number of diperoxides **8**–**12**. As can be seen from Table 2, a pronounced selective effect is observed on the myelocytic (K562) and lymphocytic (Jurkat, HL60) cell lines, in comparison with the cytotoxicity of the studied compounds to a cell culture of monocytic origin (U937). The lowest cytotoxicity with respect to the studied tumor cultures was demonstrated by symmetric diperoxides with **13** dibutyl and **14** adamantane substituents.

## 3. Materials and Methods 

### 3.1. Chemistry 

All reactions were performed at room temperature in air in round-bottom flasks equipped with a magnetic stir bar. The NMR spectra were recorded on a Bruker Avance 500 spectrometer at 500.17 MHz for ^1^H and 125.78 MHz for ^13^C according to standard Bruker procedures. CDCl_3_ was used as the solvent and tetramethylsilane as the internal standard. The mixing time for the NOESY (Nuclear Overhauser Effect SpectroscopY) experiments was 0.3 sec. Mass spectra were recorded on a Bruker Autoflex III MALDI TOF/TOF (Matrix Assisted Laser Desorption/Ionization) instrument with α-cyano-4-hydroxycinnamic acid as a matrix. Samples were prepared by the dried droplet method. C, H, and S were quantified by a Carlo Erba 1108 analyzer. The oxygen content was determined on a Carlo Erba 1108 analyzer. The progress of reactions was monitored by TLC on Sorbfil (PTSKh-AF-A) plates, with a 5:1 hexane:EtOAc mixture as the eluent and visualized with I2 vapor. For column chromatography, silica gel MACHEREY-NAGEL (0.063–0.2 mm) was used. 

The synthesis of the pentaoxacanes **1**–**7** was as reported in the literature [51]. The synthesis of the heptaoxadispiroalkanes **15**–**17** was also as reported in the literature [48]. THF was freshly distilled over LiAlH_4_. Hydrogen sulfide was obtained by the action of sodium hydrogen sulfate on hydrochloric acid.

#### 3.1.1. Reactions of Pentaoxacanes with Hydrogen Sulfide in the Presence of a Catalyst, Sm(NO_3_)_3_·6H_2_O

General procedure: A calcined and argon-filled Schlenk vessel equipped with a magnetic stir bar was charged with THF (5 mL), Sm(NO_3_)_3_·6H_2_O (0.5 mmol), and pentaoxacanes (10 mmol). The mixture was stirred at 20 °C for 1 h. Next, the hydrogen sulfide obtained by in situ was added while continuously bubbling for 1.5 h to the mixture, which was stirred for 5 h at 20 °C. After completion of the reaction, H_2_O (5 mL) and CH_2_Cl_2_ (5 mL) were added. The organic layer was separated, dried (anhydrous MgSO_4_), and concentrated to isolate products stable during storage at room temperature. Products of the reaction were purified by column chromatography on SiO_2_ using 10:1 PE:Et_2_O as the eluent. The progress of reactions was monitored by TLC, with a 5:1 hexane:EtOAc mixture as the eluent; visualization was performed with I_2_ vapor. ^1^H NMR and ^13^C NMR spectra of all new compounds are in the supplementary file.

*6,7,11,12-tetraoxa-9-thiaspiro[4.7]dodecane* (8), colorless oil; 0.19 g (98% yield), retention factors (R_f_ ) 0.74 (PE/Et_2_O = 10/1). ^1^H NMR (400 MHz, CDCl_3_, 25 °C): δ = 1.43–1.58 (m, 4H, CH_2_), 1.78–1.99 (m, 4H, CH_2_), 5.18–5.22 (m, 4H, CH_2_). ^13^C NMR (100 MHz, CDCl_3_, 25 °C): δ = 22.4, 24.5, 25.3, 29.7, 29.5, 33.0, 81.8, 81.9, 82.3, 110.1, 110.5. MALDI TOF/TOF, m/z: 191 [M-H]^+^. Anal. calcd. for C_7_H_12_O_4_S: C, 43.74; H, 6.29; S, 16.68%. Found C, 43.72; H, 6.27; S, 16.66%.

*7,8,12,13-tetraoxa-10-thiaspiro[5.7]tridecane* (9), colorless oil; 0.18 g (90% yield), R_f_ 0.76 (PE/Et_2_O = 10/1). ^1^H NMR (400 MHz, CDCl_3_, 25 °C): δ = 1.45–1.62 (m, 6H, CH_2_), 1.74–1.90 (m, 4H, CH_2_), 5.20 (s, 4H, CH_2_). ^13^C NMR (100 MHz, CDCl_3_, 25 °C): δ = 22.4, 25.3, 24.9, 25.4, 29.5, 29.8, 81.8, 110.1, 110.5. MALDI TOF/TOF, m/z: 205 [M-H]^+^. Anal. calcd. for C_8_H_14_O_4_S: C, 46.59; H, 6.84; S, 15.54%. Found C, 46.58; H, 6.82; S, 15.52%.

*1,2,6,7-tetraoxa-4-thiaspiro[7.11]nonadecane* (10), colorless oil; 0.25 g (85% yield), R_f_ 0.78 (PE/Et_2_O = 10/1). ^1^H NMR (400 MHz, CDCl_3_, 25 °C): δ = 1.27–1.81 (m, 22H, CH_2_), 5.17–5.20 (m, 4H, CH_2_). ^13^C NMR (100 MHz, CDCl_3_, 25 °C): δ = 19.3, 21.8, 22.2, 22.3, 22.6, 24.2, 24.6, 24.7, 25.9, 26.0, 26.1, 26.2, 26.9, 82.4, 83.6, 113.9. MALDI TOF/TOF, m/z: 289 [M-H]^+^. Anal. calcd. for C_14_H_26_O_4_S: C, 57.90; H, 9.02; S, 11.04%. Found C, 57.88; H, 9.00; S, 11.01%.

*3-hxyl-3-methyl-1,2,4,5,7-tetraoxathiocane* (11), colorless oil; 0.19 g (80% yield), R_f_ 0.73 (PE/Et_2_O = 10/1). ^1^H NMR (400 MHz, CDCl_3_, 25 °C): δ = 0.89–0.92 (m, 3H, CH_3_), 1.28–1.75 (m, 13H, CH_2_), 4.81–5.29 (m, 4H, CH_2_). ^13^C NMR (100 MHz, CDCl_3_, 25 °C): δ = 14.1, 18.9, 22.5, 23.9, 24.1, 29.4, 31.6, 33.9, 82.5, 83.7, 111.4. MALDI TOF/TOF, m/z: 235 [M-H]^+^. Anal. calcd. for C_10_H_20_O_4_S: C, 50.82; H, 8.53; S, 13.57%. Found C, 50.80; H, 8.51; S, 13.55%.

*3-butyl-3-ethyl-1,2,4,5,7-tetraoxathiocane* (12), colorless oil; 0.19 g (84% yield), R_f_ 0.75 (PE/Et_2_O = 10/1). ^1^H NMR (400 MHz, CDCl_3_, 25 °C): δ = 0.89–0.94 (m, 6H, CH_3_), 1.32–1.33 (m, 4H, CH_2_), 1.66–1.74 (m, 4H, CH_2_), 5.00–5.26 (m, 4H, CH_2_). ^13^C NMR (100 MHz, CDCl_3_, 25 °C): δ = 7.9, 13.9, 22.4, 22.8, 25.5, 25.6, 28.5, 29.6, 81.4, 81.6, 113.7, 113.8. MALDI TOF/TOF, m/z: 221 [M-H]^+^. Anal. calcd. for C_9_H_18_O_4_S: C, 48.63; H, 8.16; S, 14.42%. Found C, 48.61; H, 8.14; S, 14.40%.

*3,3-dibutyl-1,2,4,5,7-tetraoxathiocane* (13), colorless oil; 0.22 g (87% yield), R_f_ 0.74 (PE/Et_2_O = 10/1). ^1^H NMR (400 MHz, CDCl_3_, 25 °C): δ = 0.92–0.94 (m, 6H, CH_3_), 1.27–1.75 (m, 12H, CH_2_), 4.97–5.31 (m, 4H, CH_2_). ^13^C NMR (100 MHz, CDCl_3_, 25 °C): δ = 7.9, 13.9, 22.8, 25.6, 25.7, 25.9, 29.1, 29.3, 29.8, 81.7, 82.4, 83.6, 113.3, 113.6. MALDI TOF/TOF, m/z: 249 [M-H]^+^. Anal. calcd. for C_11_H_22_O_4_S: C, 52.77; H, 8.86; S, 12.81%. Found C, 52.75; H, 8.85; S, 12.80%.

*3-(adamantyl-2-yl)-1,2,4,5,7-tetraaoxatioocane* (14), colorless oil; 0.23 g (89% yield), R_f_ 0.76 (PE/Et_2_O = 10/1). ^1^H NMR (400 MHz, CDCl_3_, 25 °C): δ = 1.67–1.71 (m, 6H, CH_2_), 1.88 (s, 1H, CH), 2.01–2.03 (m, 4H, CH_2_), 2.33–2.38 (m, 3H, CH, CH_2_), 5.21 (d, 4H, J = 4 Hz, CH_2_). ^13^C NMR (100 MHz, CDCl_3_, 25 °C): δ = 26.9, 27.0, 27.1, 31.2, 31.5, 33.7, 37.7, 37.1, 81.7, 112.1, 112.6. MALDI TOF/TOF, m/z: 257 [M-H]^+^. Anal. calcd. for C_12_H_18_O_4_S: C, 55.79; H, 7.02; S, 12.41%. Found C, 55.77; H, 7.00; S, 12.40%.

#### 3.1.2. Reactions Heptaoxadispiroalkanes with Hydrogen Sulfide in Presence of a Catalyst, Sm(NO_3_)_3_·6H_2_O

General procedure: A calcined and argon-filled Schlenk vessel equipped with a magnetic stir bar was charged with THF (5 mL), Sm(NO_3_)_3_·6H_2_O (0.5 mmol), and heptaoxadispiroalkanes (10 mmol). The mixture was stirred at 20 °C for 1 h. Next, the hydrogen sulfide obtained in situ was added while continuously bubbling for 1.5 h to the mixture, which was stirred for 5 h at 20 °C. After completion of the reaction, H_2_O (5 mL) and CH_2_Cl_2_ (5 mL) were added. The organic layer was separated, dried (anhydrous MgSO_4_), and concentrated to isolate products stable during storage at room temperature. Products of the reaction were purified by column chromatography on SiO_2_ using 10:1 PE:Et_2_O as the eluent. The progress of reactions was monitored by TLC, with a 5:1 hexane:EtOAc mixture as the eluent; visualization was performed with I_2_ vapor. 

*6,7,13,14,18,19-hexaoxa-16-thiadispiro[4.2.4^8^.7^5^]nonadecane* (15), colorless oil; 0.29 g (87% yield), R_f_ 0.79 (PE/Et_2_O = 10/1). ^1^H NMR (400 MHz, CDCl_3_, 25 °C): δ = 1.73–1.80 (m, 4H, CH_2_), 1.93–2.09 (m, 4H, CH_2_), 5.13–5.25 (m, 4H, CH_2_). ^13^C NMR (100 MHz, CDCl_3_, 25 °C): δ = 24.5, 24.6, 33.1, 33.3, 33.4, 33.8, 33.9, 81.9, 82.5, 120.3. MALDI TOF/TOF, m/z: 291 [M-H]^+^. Anal. calcd. for C_12_H_20_O_6_S: C, 49.30; H, 6.90; S, 10.97%. Found C, 49.28; H, 6.89; S, 10.95%.

*3,12-dimethyl-7,8,15,16,20,21-hexaoxa-18-thiadispiro[5.2.5^9^.7^6^]henicosane* (16), colorless oil; 0.29 g (83% yield), R_f_ 0.79 (PE/Et_2_O = 10/1). ^1^H NMR (400 MHz, CDCl_3_, 25 °C): δ = 0.93–0.94 (m, 6H, CH_3_), 1.20–1.26, and 1.44–1.57 (m, 8H, CH_2_), 1.60–1.64 and 2.16–2.25 (m, 8H, CH_2_), 1.99–2.00 (m, 2H, CH), 5.18–5.23 (m, 4H, CH_2_). ^13^C NMR (100 MHz, CDCl_3_, 25 °C): δ = 21.3, 21.4, 22.7, 29.1, 29.2, 29.3, 29.4,29.8, 30.5, 30.6, 30.7, 31.6, 31.7, 33.1, 81.8, 81.9, 110.1, 111.1. MALDI TOF/TOF, m/z: 347 [M-H]^+^. Anal. calcd. for C_16_H_28_O_6_S: C, 55.15; H, 8.10; S, 9.20%. Found C, 55.13; H, 8.08; S, 9.17%.

*8,9,17,18,22,23-hexaoxa-20-thiadispiro[6.2.6^10^.7^7^]tricosane* (17), colorless oil; 0.29 g (85% yield), R_f_ 0.80 (PE/Et_2_O = 10/1). ^1^H NMR (400 MHz, CDCl_3_, 25 °C): δ = 1.58–1.73 (m, 16H, CH_2_), 1.86–2.04 (m, 8H, CH_2_), 5.13–5.31 (m, 4H, CH_2_). ^13^C NMR (100 MHz, CDCl_3_, 25 °C): δ = 22.7, 22.8, 29.8, 29.9, 30.2, 30.4, 32.4, 32.8, 32.9, 81.8, 82.5, 115.2, 116.2. MALDI TOF/TOF, m/z: 347 [M-H]^+^. Anal. calcd. for C_16_H_28_O_6_S: C, 55.15; H, 8.10; S, 9.20%. Found C, 55.14; H, 8.08; S, 9.18%.

### 3.2. Biology

#### 3.2.1. Cell Culturing 

Cells (Jurkat, K562, U937, HeLa, HEK293, and normal fibroblasts) were purchased from Russian Cell Culture Collection (Institute of Cytology of the Russian Academy of Sciences) and cultured according to standard mammalian tissue culture protocols and sterile technique. Human cell lines HEK293 and HeLa were obtained from the HPA Culture Collections (U.K.). All cell lines used in the study were tested and shown to be free of mycoplasma and viral contamination. 

HEK293, HeLa cell lines, and fibroblasts were cultured as monolayers and maintained in Dulbecco’s modified eagle’s medium (DMEM, Gibco BRL) supplemented with 10% fetal bovine serum (FBS) and 1% penicillin-streptomycin solution at 37 °C in a humidified incubator under a 5% CO_2_ atmosphere.

Cells were maintained in RPMI (Roswell Park Memorial Institute medium) 1640 (Jurkat, K562, U937) (Gibco) supplemented with 4 mM glutamine, 10% FBS (Sigma), and 100 units/mL penicillin-streptomycin (Sigma). All types of cells were grown in an atmosphere of 5% CO_2_ at 37 °C. The cells were subcultured at 2- to 3-day intervals. Adherent cells (HEK293, HeLa, fibroblasts) were suspended using trypsin/EDTA (Ethylenediaminetetraacetic acid) and counted after they reached 80% confluency. Cells were then seeded in 24 well plates at 5 × 10^4^ cells per well and incubated overnight. Jurkat, K562, and U937 cells were subcultured in 2-day intervals with a seeding density of 1 × 10^5^ cells per 24 well plates in RPMI with 10% FBS.

#### 3.2.2. Cytotoxicity Assay 

Viability (live/dead) assessment was performed by staining cells with 7-aminoactinomycin D (7-AAD) (Biolegend). After treatment, cells were harvested, washed 1 to 2 times with phosphate-buffered saline (PBS), and centrifuged at 400× *g* for 5 min. Cell pellets were resuspended in 200 µL of flow cytometry staining buffer (PBS without Ca^2+^ and Mg^2+^, 2,5% FBS) and stained with 5 µL of 7-AAD staining solution for 15 min at room temperature in the dark. Samples were acquired on the NovoCyteTM 2000 FlowCytometry System (ACEA) equipped with a 488 nm argon laser. Detection of 7-AAD emission was collected through a 675/30 nm filter in the FL4 channel.

## 4. Conclusions

For the first time, an approach was developed that allows for the selective synthesis of new classes of stable tetraoxathiaspiroalkanes, tetraoxathiocanes, and hexaoxathiadispiroalkanes by reactions of pentaoxaspiroalkanes, pentaoxacanes, and heptaoxadispiroalkanes with hydrogen sulfide in the presence of lanthanide catalysts (Sm(NO_3_)_3_^.^6H_2_O, Ho(NO_3_)_3_∙5H_2_O, TbCl_3_∙6H_2_O, DyCl_3_∙6H_2_O, NdCl_3_, La(NO_3_)_3_). In addition, we found that the synthesized S-containing di- and triperoxides exhibit high cytotoxic activity against Jurkat, K562, U937, HL60 tumor cultures and fibroblasts.

## Figures and Tables

**Table 1:** Optimization of the reaction conditions for the synthesis 7,8,12,13-tetraoxa-10-thiaspiro[5.7]tridecane (**8**).

Entry	[M]	Solvent	Yield * of 8%
1	Sm(NO_3_)_3_·6H_2_O	THF	98
2	Sm(NO_3_)_3_·6H_2_O	CH_2_Cl_2_	85
3	Sm(NO_3_)_3_·6H_2_O	Et_2_O	79
4	Sm(NO_3_)_3_·6H_2_O	C_6_H_12_	15
5	Sm(NO_3_)_3_·6H_2_O	EtOAc	10
6	Sm(NO_3_)_3_·6H_2_O	C_2_H_5_OH	7
7	Ho(NO_3_)_3_ 5H_2_O	THF	84
8	TbCl_3_ 6H_2_O	THF	72
9	DyCl_3_ 6H_2_O	THF	67
10	NdCl_3_ 6H_2_O	THF	61
11	La(NO_3_)_3_·6H_2_O	THF	58

* Experimental conditions: **1**:[M] molar ratio of 1:0.05; 20 °C; 6 h; 5 mL solvent.

**Table 2:** Cytotoxic activities in vitro of compounds **8**–**14** and **18**–**20** measured on tumor cell cultures (Jurkat, K562, U937, and HL60, fibroblasts) (µM).

Compound	Jurkat (IC_50_, µM)	K562 (IC_50_, µM)	HL60 (IC_50_, µM)	U937 (IC_50_, µM)	Fibroblasts (IC_50_, µM)
8	5.26 ± 0.57	7.15 ± 0.64	4.59 ± 0.38	24.13 ± 1.87	118.61 ± 8.74
9	4.91 ± 0.43	6.83 ± 0.59	4.14 ± 0.34	21.17 ± 2.11	97.88 ± 6.81
10	3.52 ± 0.31	5.77 ± 0.46	2.67 ± 0.21	15.24 ± 1.26	81.42 ± 5.12
12	4.45 ± 0.49	6.29 ± 0.57	3.91 ± 0.33	19.89 ± 1.57	85.93 ± 5.47
13	10.21 ± 0.87	14.37 ± 0.96	8.56 ± 0.69	35.24 ± 2.65	142.17 ± 9.76
14	9.61 ± 0.79	11.97 ± 0.91	8.22 ± 0.74	32.81 ± 2.89	129.23 ± 8.92
18	17.11 ± 1.24	21.75 ± 1.59	14.96 ± 0.97	46.67 ± 3.76	188.36 ± 12.91
19	2.81 ± 0.37	4.37 ± 0.31	2.24 ± 0.29	11.79 ± 0.99	79.17 ± 5.41
20	23.94 ± 1.67	28.26 ± 1.48	19.61 ± 1.12	65.81 ± 4.84	195.87 ± 14.67

**IC_50_**, or the concentration of half-maximal inhibition, is an indicator of the effectiveness of a ligand in inhibiting biochemical or biological interaction.

## Supplementary Materials

The following are available online: ^1^H NMR and ^13^C NMR spectra of all new compounds.
