# Supplementary material for: First Example of Catalytic Synthesis of Cyclic S-Containing Di- and Triperoxides
Source: Molecules. 2020 Apr 18;25(8):1874. doi: 10.3390/molecules25081874 (PMC7221664; doi:10.3390/molecules25081874)
Supplement: Supplementary file 1 [file molecules-25-01874-s001.pdf]

*molecules*

*Supporting information*

# First Example of Catalytic Synthesis of Cyclic S-Containing Di- and Triperoxides

**Nataliya Makhmudiyarova *, Irina Ishmukhametova, Lilya Dzhemileva *, Vladimir D'yakonov, Askhat Ibragimov and Usein Dzhemilev**

Institute of Petrochemistry and Catalysis, Russian Academy of Sciences, 141 Prospekt Oktyabrya, Ufa 450075, Russian Federation; E-mails: iuliania93@mail.ru (I.I.); dzhemilev@mail.ru (L.D.); DyakonovVA@gmail.com (V.D.) a.ibragimov@mail.ru (A.I.); dzhemilev@anrb.ru (U.D.)

* Correspondence: natali-mnn@mail.com (N.M.); dzhemilev@mail.ru (L.D.); Tel.: +7-917-368-65-59 (N.M.); Tel.: +7-917-458-47-29 (L.D.).

Received: 23 March 2020; Accepted: 15 April 2020; Published: date

**-Table of Contents-**


| | | |
|---|---|---|
| 1. | General Information | page 1 |
| 2. | Copy of NMR spectra | page 4 |
| 3. | References | page 14 |


## 1. General Information

### *1.1. General Remarks*

All reactions were performed at room temperature in air in round-bottom flasks equipped with a magnetic stir bar. The NMR spectra were recorded on a Bruker Avance 500 spectrometer at 500.17 MHz for $^{1}H$ and 125.78 MHz for $^{13}C$ according to standard Bruker procedures. $CDCl_3$ was used as the solvent, and tetramethylsilane as the internal standard. The mixing time for the NOESY experiments was 0.3 s. Mass spectra were recorded on a Bruker Autoflex III MALDI TOF/TOF instrument with $\alpha$-cyano-4-hydroxycinnamic acid as a matrix. Samples were prepared via the dried droplet method. C, H, and S were quantified using a Carlo Erba 1108 analyzer. The oxygen content was determined on a Carlo Erba 1108 analyzer. The progress of reactions was monitored by TLC on Sorbfil (PTSKh-AF-A) plates, with a 5:1 hexane:EtOAc mixture as the eluent and visualized with $I_2$ vapor. For column chromatography, silica gel MACHEREY-NAGEL (0.063–0.2 mm) was used.

The synthesis of the pentaoxacanes **(1–7)** and heptaoxadispiroalkanes (**15–17)** was as reported in the literature [1,2]. THF was freshly distilled over $LiAlH_4$. Hydrogen sulfide was obtained by the action of sodium hydrogen sulfate on sulfuric acid.

### *1.2. Cell Culturing*

Human cancer cell line HeLa was obtained from the HPA Culture Collections (U.K.). Cells (Jurkat, K562, U937, HL60, and fibroblasts) were purchased from Russian Cell Culture Collection (Institute of Cytology of the Russian Academy of Sciences) and cultured according to standard protocols and sterile technique. The cell lines were found to be free of viral contamination and mycoplasma. Cells were maintained in RPMI 1640 (Jurkat, K562, U937, HL60, and fibroblasts; Gibco) supplemented with 4 μM glutamine, 10% FBS (Sigma), and 100 units/mL penicillin-streptomycin (Sigma). All types of cells were grown in an atmosphere of 5% $CO_2$ at 37 °C. The cells were subcultured in 2–3 days intervals. Cells were then seeded in 24 well plates at $5 \times 10^4$ cells per well and incubated overnight. Jurkat, K562, U937, HL60, and fibroblasts cells were subcultured in 2-day intervals with a seeding density of $1 \times 10^5$ cells per 24 well plates in RPMI with 10% FBS.

### *1.3. Cytotoxicity Assay*

Viability (Live/dead) assessment was performed by staining cells with 7-Aminoactinomycin D (7-AAD; Biolegend). After treatment, cells were harvested, washed 1–2 times with phosphate-buffered saline (PBS), and centrifuged at 400 ×*g* for 5 min. Cell pellets were resuspended in 200 μL of flow cytometry staining buffer (PBS without $Ca^{2+}$ and $Mg^{2+}$, 2.5% FBS) and stained with 5 μL of 7-AAD staining solution for 15 min at room temperature in the dark. Samples were acquired on NovoCyte TM 2000 Flow Cytometry System (ACEA) equipped with 488 nm argon laser. We detected 7-AAD emission through a 675/30 nm filter in the FL4 channel.

### *1.4. Reactions of Pentaoxacanes with Hydrogen Sulfide in the Presence of a Catalyst, $Sm(NO_3)_3{\cdot}6H_2O$.*

General procedure: A calcined and argon-filled Schlenk vessel equipped with a magnetic stir bar was charged with THF (5 mL), $Sm(NO_3)_3{\cdot}6H_2O$ (0.5 mmol), and pentaoxacanes[1] (10 mmol). The mixture was stirred at 20 °C for 1 h. Next, the hydrogen sulfide obtained in situ was added, continuously bubbling the mixture, which was stirred for 3 h at 20 °C. After completion of the reaction $H_2O$ (5 mL) and $CH_2Cl_2$ (5 mL) were added. The organic layer was separated, dried (anhydrous $MgSO_4$), and concentrated to isolate products stable during storage at room temperature. Products of the reaction were purified by column chromatography on $SiO_2$ using 10:1 PE:$Et_2O$ as the eluent. The progress of reactions was monitored by TLC, with a 5:1 hexane:EtOAc mixture as the eluent; visualization was performed with $I_2$ vapor.

*6,7,11,12-Tetraoxa-9-thiaspiro[4.7]dodecane* (**8**), Colorless oil; 0.19 g (98% yield), $R_f$ 0.74 (PE/$Et_2O$ = 10/1). $^1H$ NMR (400 MHz, $CDCl_3$, 25 °C): δ = 1.43–1.58 (m, 4H, $CH_2$), 1.78–1.99 (m, 4H, $CH_2$), 5.18–5.22 (m, 4H, $CH_2$). $^{13}C$ NMR (100 MHz, $CDCl_3$, 25 °C): δ = 22.4, 24.5, 25.3, 29.7, 29.5, 33.0, 81.8, 81.9, 82.3, 110.1, 110.5. MALDI TOF/TOF, m/z: 191 $[M-H]^+$. Anal. calcd. for $C_7H_{12}O_4S$: C, 43.74; H, 6.29; S, 16.68%. Found C, 43.72; H, 6.27; S, 16.66%.

*7,8,12,13-Tetraoxa-10-thiaspiro[5.7]tridecane* (**9**), Colorless oil; 0.18 g (90% yield), $R_f$ 0.76 (PE/$Et_2O$ = 10/1). $^1H$ NMR (400 MHz, $CDCl_3$, 25 °C): δ = 1.45–1.62 (m, 6H, $CH_2$), 1.74–1.90 (m, 4H, $CH_2$), 5.20 (s, 4H, $CH_2$). $^{13}C$ NMR (100 MHz, $CDCl_3$, 25 °C): δ = 22.4, 25.3, 24.9, 25.4, 29.5, 29.8, 81.8, 110.1, 110.5. MALDI TOF/TOF, m/z: 205 $[M-H]^+$. Anal. calcd. for $C_8H_{14}O_4S$: C, 46.59; H, 6.84; S, 15.54%. Found C, 46.58; H, 6.82; S, 15.52%.

*1,2,6,7-Tetraoxa-4-thiaspiro[7.11]nonadecane* (**10**), Colorless oil; 0.25 g (85% yield), $R_f$ 0.78 (PE/$Et_2O$ = 10/1). $^1H$ NMR (400 MHz, $CDCl_3$, 25 °C): δ = 1.27–1.81 (m, 22H, $CH_2$), 5.17–5.20 (m, 4H, $CH_2$). $^{13}C$ NMR (100 MHz, $CDCl_3$, 25 °C): δ = 19.3, 21.8, 22.2, 22.3, 22.6, 24.2, 24.6, 24.7, 25.9, 26.0, 26.1, 26.2, 26.9, 82.4, 83.6, 113.9. MALDI TOF/TOF, m/z: 289 $[M-H]^+$. Anal. calcd. for $C_{14}H_{26}O_4S$: C, 57.90; H, 9.02; S, 11.04%. Found C, 57.88; H, 9.00; S, 11.01%.

*3-Hexyl-3-methyl-1,2,4,5,7-tetraoxathiocane* (**11**), Colorless oil; 0.19 g (80% yield), $R_f$ 0.73 (PE/$Et_2O$ = 10/1). $^1H$ NMR (400 MHz, $CDCl_3$, 25 °C): δ = 0.89–0.92 (m, 3H, $CH_3$), 1.28–1.75 (m, 13H, $CH_2$), 4.81–5.29 (m, 4H, $CH_2$). $^{13}C$ NMR (100 MHz, $CDCl_3$, 25 °C): δ = 14.1, 18.9, 22.5, 23.9, 24.1, 29.4, 31.6, 33.9, 82.5, 83.7, 111.4. MALDI TOF/TOF, m/z: 235 $[M-H]^+$. Anal. calcd. for $C_{10}H_{20}O_4S$: C, 50.82; H, 8.53; S, 13.57%. Found C, 50.80; H, 8.51; S, 13.55%.

*3-Butyl-3-ethyl-1,2,4,5,7-tetraoxathiocane* (**12**), Colorless oil; 0.19 g (84% yield), $R_f$ 0.75 (PE/$Et_2O$ = 10/1). $^1H$ NMR (400 MHz, $CDCl_3$, 25 °C): δ = 0.89–0.94 (m, 6H, $CH_3$), 1.32–1.33 (m, 4H, $CH_2$), 1.66–1.74 (m, 4H, $CH_2$), 5.00–5.26 (m, 4H, $CH_2$). $^{13}C$ NMR (100 MHz, $CDCl_3$, 25 °C): δ = 7.9, 13.9, 22.4, 22.8, 25.5, 25.6, 28.5, 29.6, 81.4, 81.6, 113.7, 113.8. MALDI TOF/TOF, m/z: 221 $[M-H]^+$. Anal. calcd. for $C_9H_{18}O_4S$: C, 48.63; H, 8.16; S, 14.42%. Found C, 48.61; H, 8.14; S, 14.40%.

*3,3-Dibutyl-1,2,4,5,7-tetraoxathiocane* (**13**), Colorless oil; 0.22 g (87% yield), $R_f$ 0.74 (PE/$Et_2O$ = 10/1). $^1H$ NMR (400 MHz, $CDCl_3$, 25 °C): δ = 0.92–0.94 (m, 6H, $CH_3$), 1.27–1.75 (m, 12H, $CH_2$), 4.97–5.31 (m, 4H, $CH_2$). $^{13}C$ NMR (100 MHz, $CDCl_3$, 25 °C): δ = 7.9, 13.9, 22.8, 25.6, 25.7, 25.9, 29.1, 29.3, 29.8, 81.7, 82.4, 83.6, 113.3, 113.6. MALDI TOF/TOF, m/z: 249 $[M-H]^+$. Anal. calcd. for $C_{11}H_{22}O_4S$: C, 52.77; H, 8.86; S, 12.81%. Found C, 52.75; H, 8.85; S, 12.80%.

*3-(Adamantyl-2-yl)-1,2,4,5,7-tetraaoxatioocane* (**14**), Colorless oil; 0.23 g (89% yield), $R_f$ 0.76 (PE/$Et_2O$ = 10/1). $^1H$ NMR (400 MHz, $CDCl_3$, 25 °C): δ = 1.67–1.71 (m, 6H, $CH_2$), 1.88 (s, 1H, CH), 2.01–2.03 (m, 4H, $CH_2$), 2.33–2.38 (m, 3H, CH, $CH_2$), 5.21 (d, 4H, J = 4 Hz, $CH_2$). $^{13}C$ NMR (100 MHz, $CDCl_3$, 25 °C): δ = 26.9, 27.0, 27.1, 31.2, 31.5, 33.7, 37.7, 37.1, 81.7, 112.1, 112.6. MALDI TOF/TOF, m/z: 257 $[M-H]^+$. Anal. calcd. for $C_{12}H_{18}O_4S$: C, 55.79; H, 7.02; S, 12.41%. Found C, 55.77; H, 7.00; S, 12.40%.

3.1.2. Reactions of Heptaoxadispiroalkanes with Hydrogen Sulfide in the Presence of a Catalyst, $Sm(NO_3)_3{\cdot}6H_2O$.

General procedure: A calcined and argon-filled Schlenk vessel equipped with a magnetic stir bar was charged with THF (5 mL), $Sm(NO_3)_3{\cdot}6H_2O$ (0.5 mmol), and heptaoxadispiroalkanes (10 mmol). The mixture was stirred at 20 °C for 1 h. Next, the hydrogen sulfide obtained in situ was added with continuous bubbling for 1.5 h to the mixture, which was stirred for 3 h at 20 °C. After completion of the reaction, $H_2O$ (5 mL) and $CH_2Cl_2$ (5 mL) were added. The organic layer was separated, dried (anhydrous $MgSO_4$), and concentrated to isolate products stable during storage at room temperature. Products of the reaction were purified by column chromatography on $SiO_2$ using 10:1 PE:$Et_2O$ as the eluent. The progress of reactions was monitored by TLC, with a 5:1 hexane:EtOAc mixture as the eluent; visualization was performed with $I_2$ vapor.

*6,7,13,14,18,19-Hexaoxa-16-thiadispiro[4.2.4⁸.7⁵]nonadecane* (**15**), Colorless oil; 0.29 g (87% yield), $R_f$ 0.79 (PE/$Et_2O$ = 10/1). $^1H$ NMR (400 MHz, $CDCl_3$, 25 °C): δ = 1.73–1.80 (m, 4H, $CH_2$), 1.93–2.09 (m, 4H, $CH_2$), 5.13–5.25 (m, 4H, $CH_2$). $^{13}C$ NMR (100 MHz, $CDCl_3$, 25 °C): δ = 24.5, 24.6, 33.1, 33.3, 33.4, 33.8, 33.9, 81.9, 82.5, 120.3. MALDI TOF/TOF, m/z: 291 $[M-H]^+$. Anal. calcd. for $C_{12}H_{20}O_6S$: C, 49.30; H, 6.90; S, 10.97%. Found C, 49.28; H, 6.89; S, 10.95%.

*3,12-Dimethyl-7,8,15,16,20,21-hexaoxa-18-thiadispiro[5.2.5⁹.7⁶]henicosane* (**16**), Colorless oil; 0.29 g (83% yield), $R_f$ 0.79 (PE/$Et_2O$ = 10/1). $^1H$ NMR (400 MHz, $CDCl_3$, 25 °C): δ = 0.93–0.94 (m, 6H, $CH_3$), 1.20–1.26 and 1.44–1.57 (m, 8H, $CH_2$), 1.60–1.64 and 2.16–2.25 (m, 8H, $CH_2$), 1.99–2.00 (m, 2H, CH), 5.18–5.23 (m, 4H, $CH_2$). $^{13}C$ NMR (100 MHz, $CDCl_3$, 25 °C): δ = 21.3, 21.4, 22.7, 29.1, 29.2, 29.3, 29.4,29.8, 30.5, 30.6, 30.7, 31.6, 31.7, 33.1, 81.8, 81.9, 110.1, 111.1. MALDI TOF/TOF, m/z: 347 $[M-H]^+$. Anal. calcd. for $C_{16}H_{28}O_6S$: C, 55.15; H, 8.10; S, 9.20%. Found C, 55.13; H, 8.08; S, 9.17%.

*8,9,17,18,22,23-Hexaoxa-20-thiadispiro[6.2.6¹⁰.7⁷]tricosane* (**17**), Colorless oil; 0.29 g (85% yield), $R_f$ 0.80 (PE/$Et_2O$ = 10/1). $^1H$ NMR (400 MHz, $CDCl_3$, 25 °C): δ = 1.58–1.73 (m, 16H, $CH_2$), 1.86–2.04 (m, 8H, $CH_2$), 5.13–5.31 (m, 4H, $CH_2$). $^{13}C$ NMR (100 MHz, $CDCl_3$, 25 °C): δ = 22.7, 22.8, 29.8, 29.9, 30.2, 30.4, 32.4, 32.8, 32.9, 81.8, 82.5, 115.2, 116.2. MALDI TOF/TOF, m/z: 347 $[M-H]^+$. Anal. calcd. for $C_{16}H_{28}O_6S$: C, 55.15; H, 8.10; S, 9.20%. Found C, 55.14; H, 8.08; S, 9.18%.

## 2. Copy of NMR Spectra

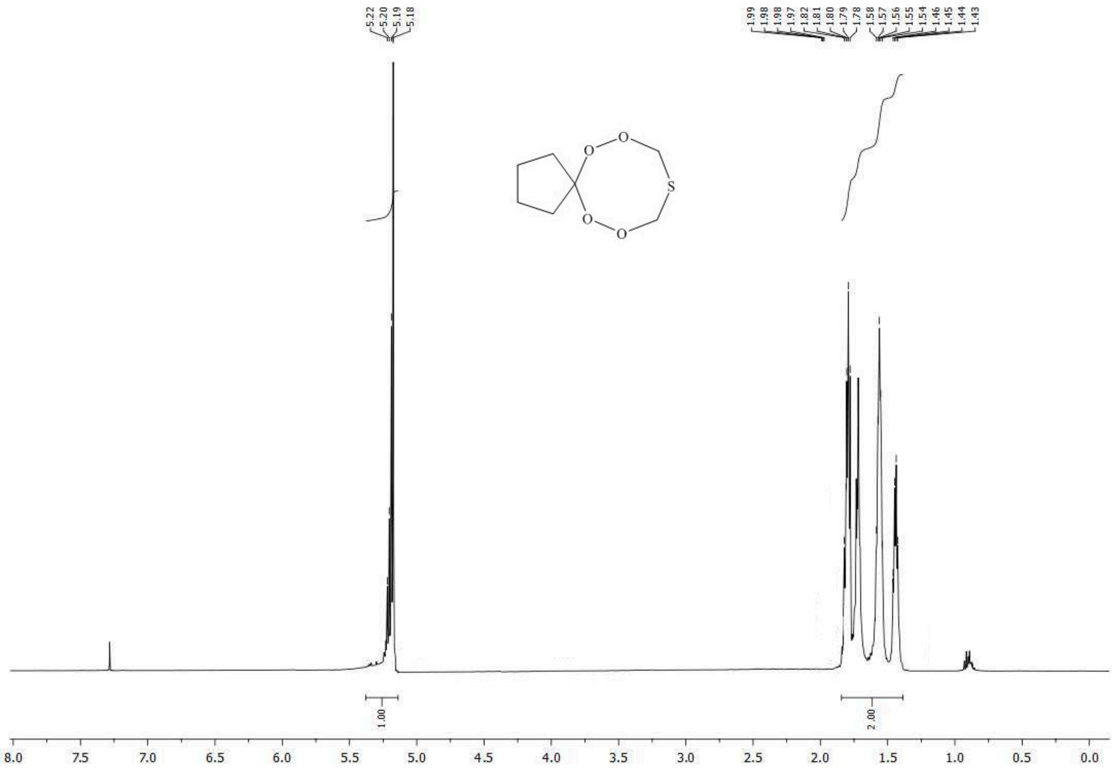


**$^1$H-NMR spectrum of 6,7,11,12-tetraoxa-9-thiaspiro[4.7]**dodecane (**8**).

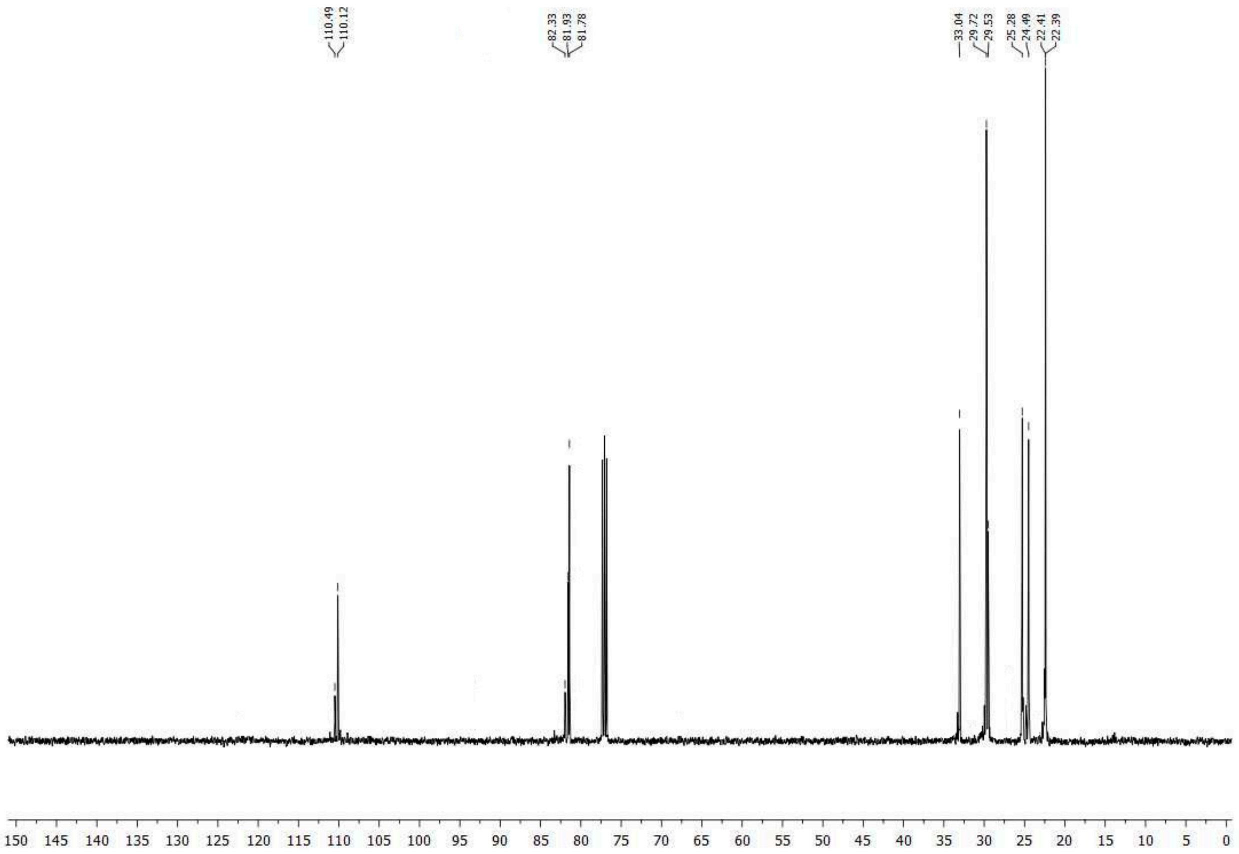


**$^{13}$C-NMR spectrum of 6,7,11,12-tetraoxa-9-thiaspiro[4.7]**dodecane (**8**).

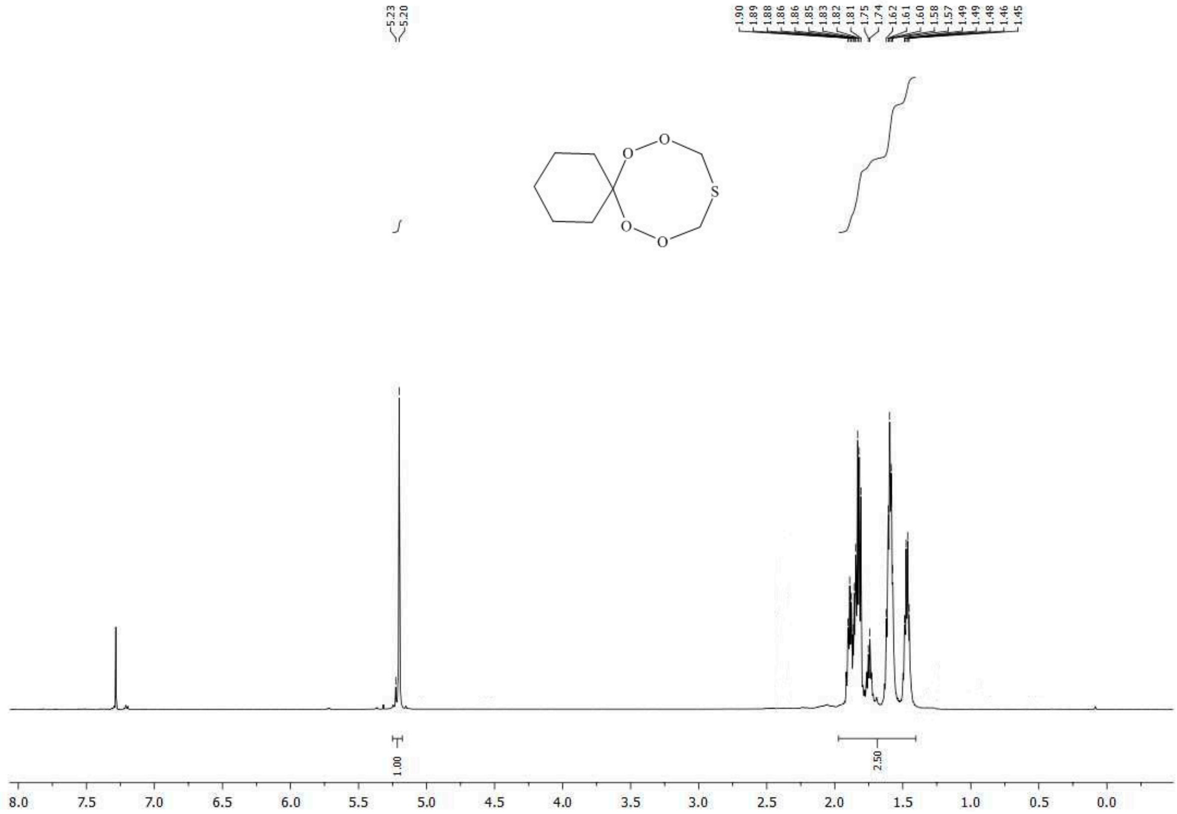


**$^{1}$H-NMR spectrum of 7,8,12,13-tetraoxa-10-thiaspiro[5.7]**tridecane (**9**).

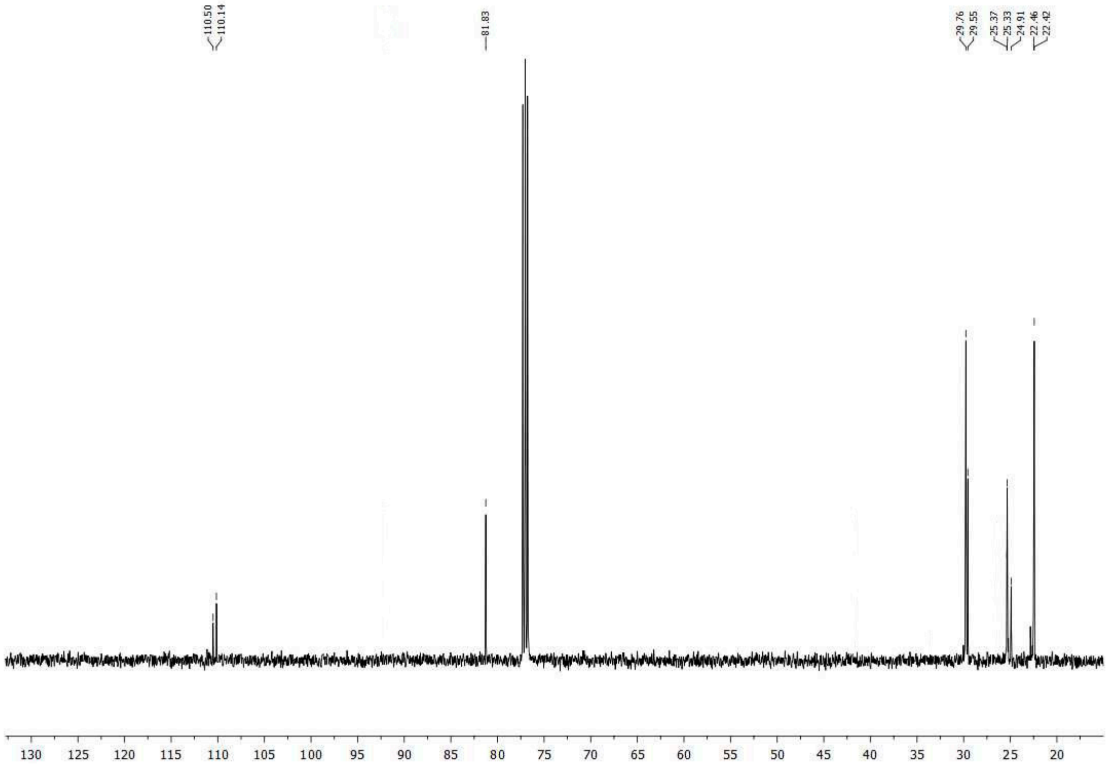


**$^{13}$C-NMR spectrum of 7,8,12,13-tetraoxa-10-thiaspiro[5.7]**tridecane (**9**).

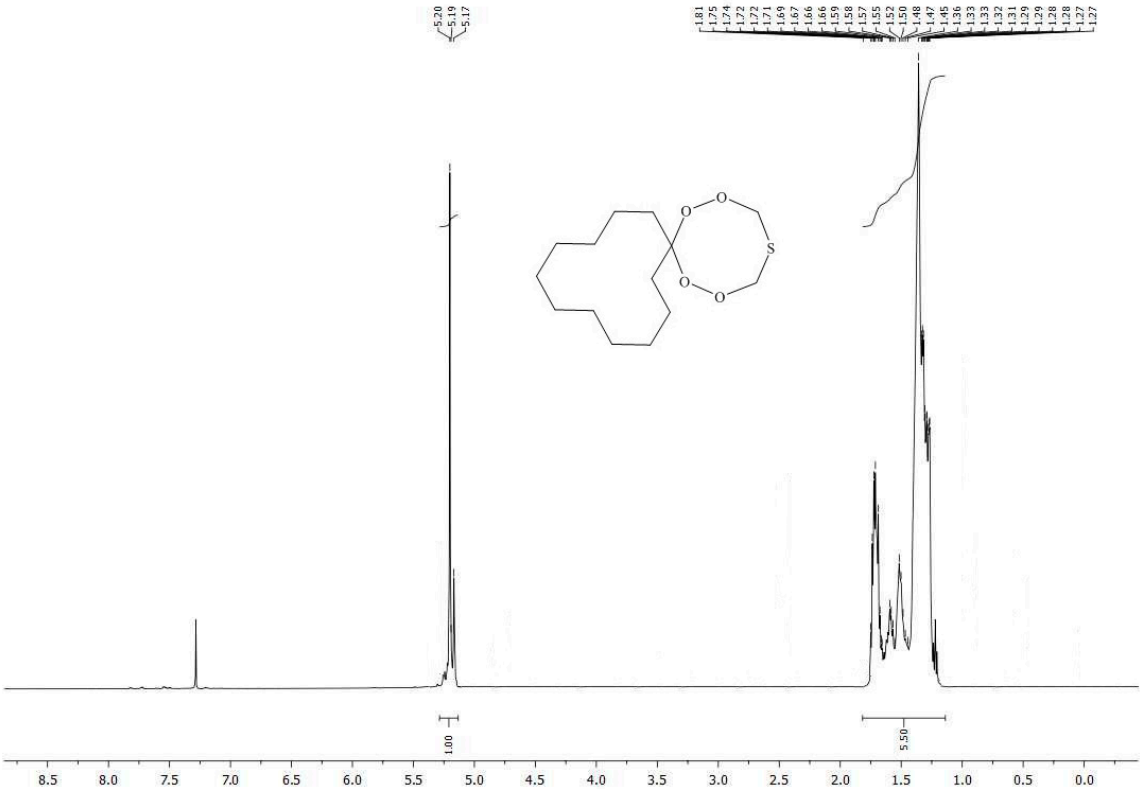


**$^{1}$H-NMR spectrum of 1,2,6,7-tetraoxa-4-thiaspiro[7.1**1]nonadecane (**10**).

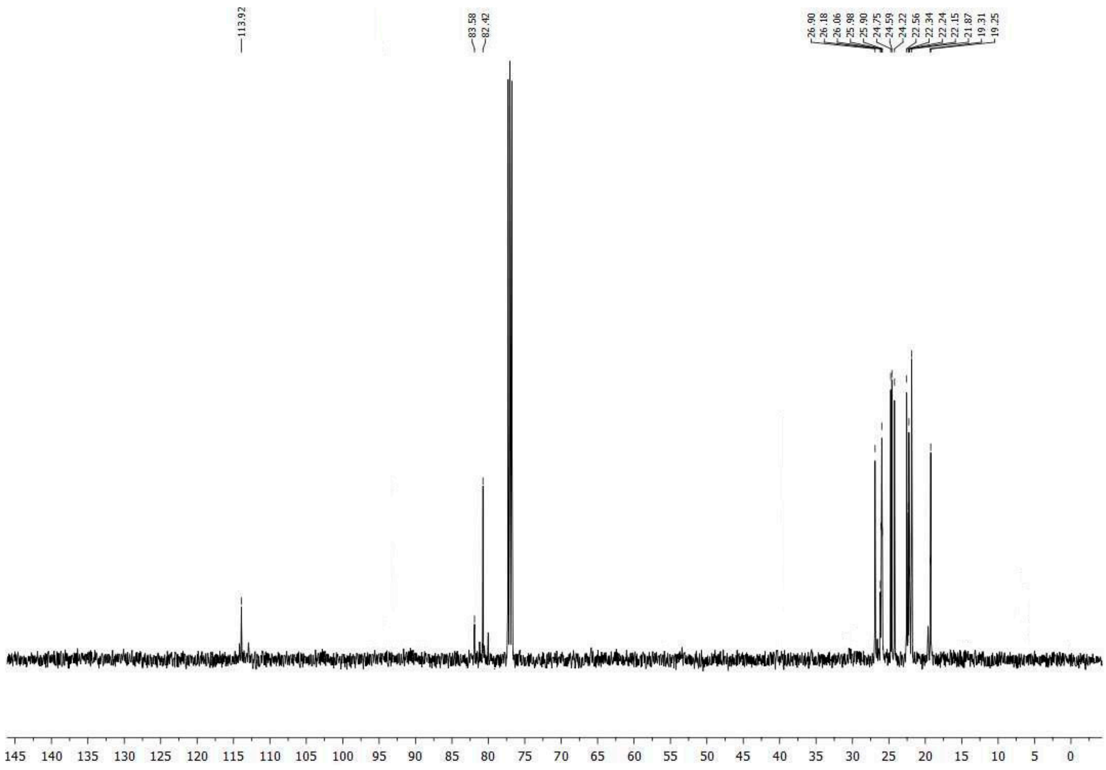


**$^{13}$C-NMR spectrum of 1,2,6,7-tetraoxa-4-thiaspiro[7.1**1]nonadecane (**10**).

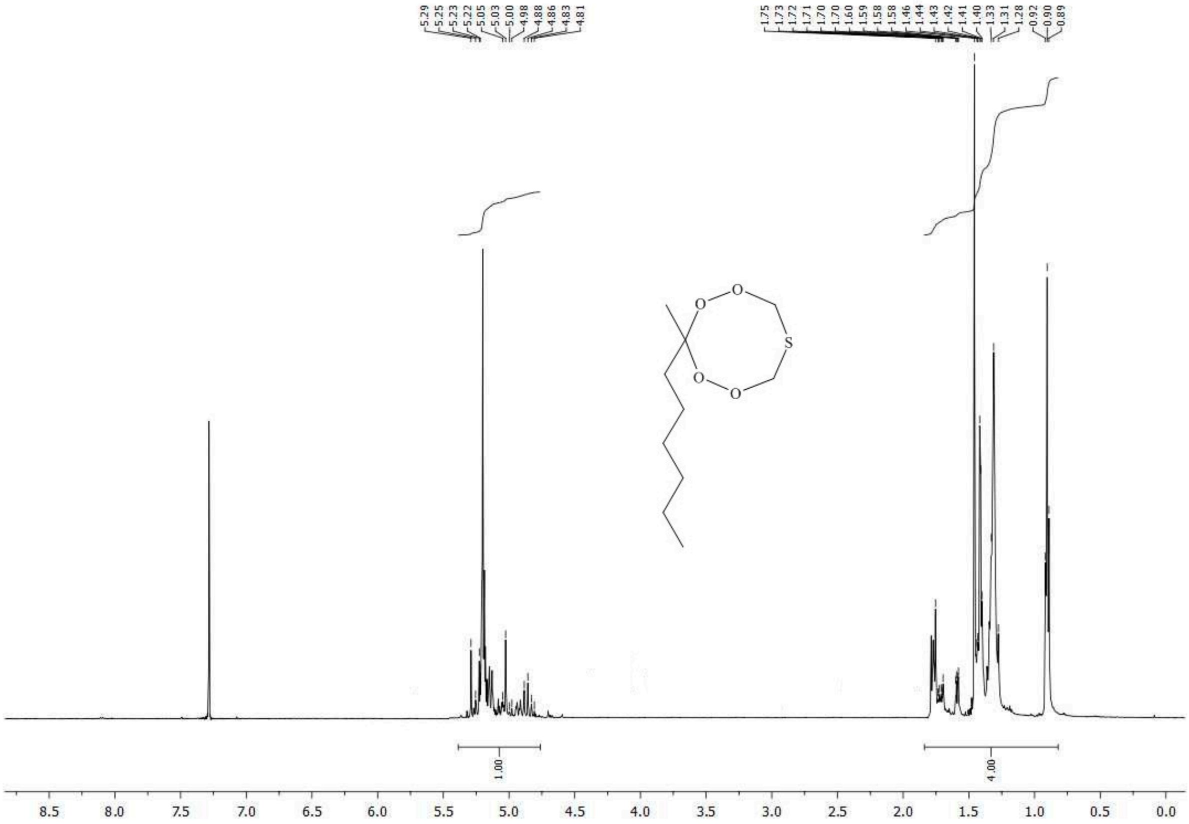


**$^{1}$H-NMR spectrum of 3-hexyl-3-methyl-1,2,4,5,7-tetraoxathiocane (11).**

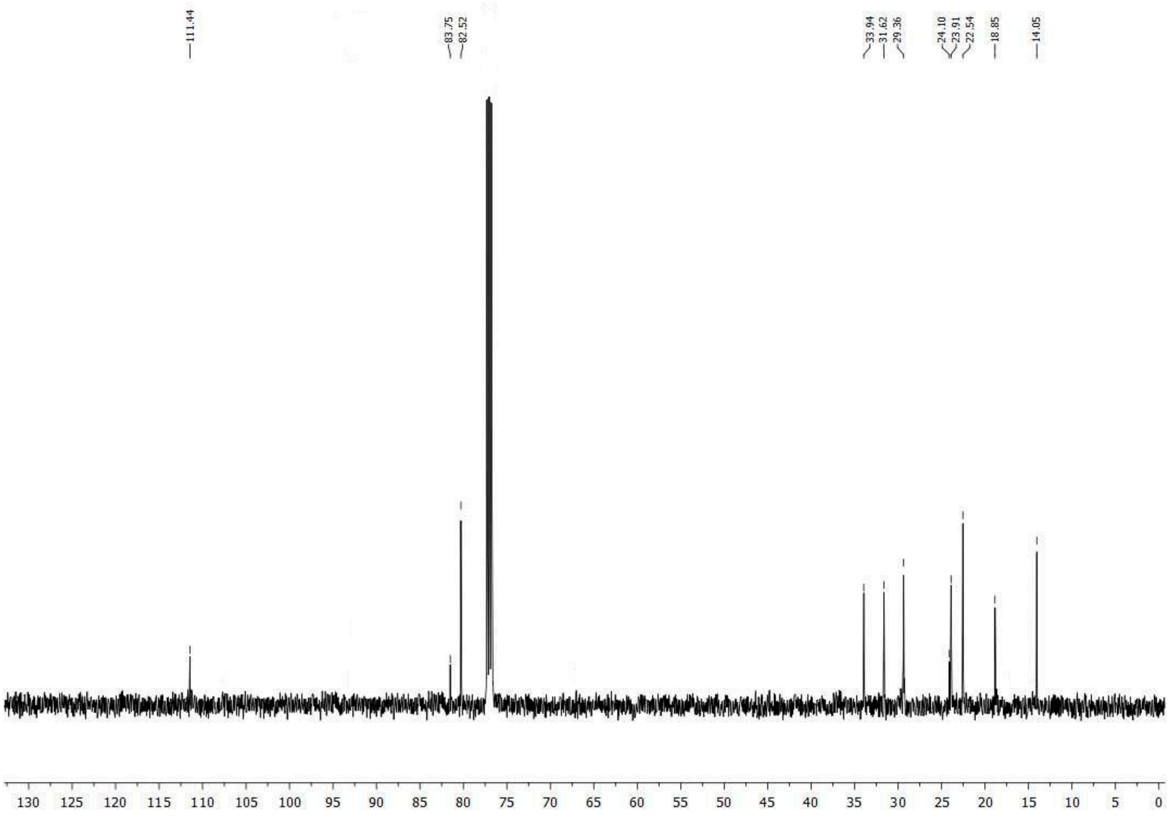


**$^{13}$C-NMR spectrum of 3-hexyl-3-methyl-1,2,4,5,7-tetraoxathiocane (11).**

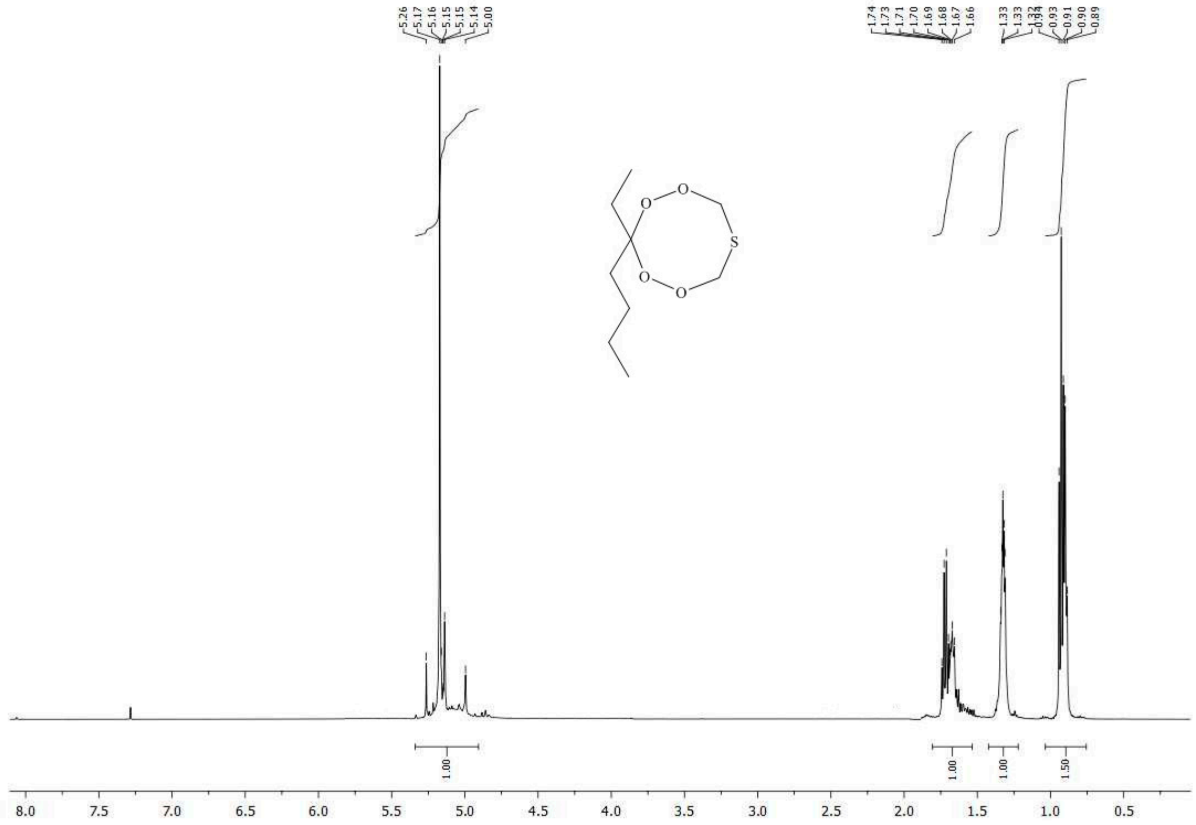


**$^{1}$H-NMR spectrum of 3-butyl-3-ethyl-1,2,4,5,7-tetraoxathiocane (12).**

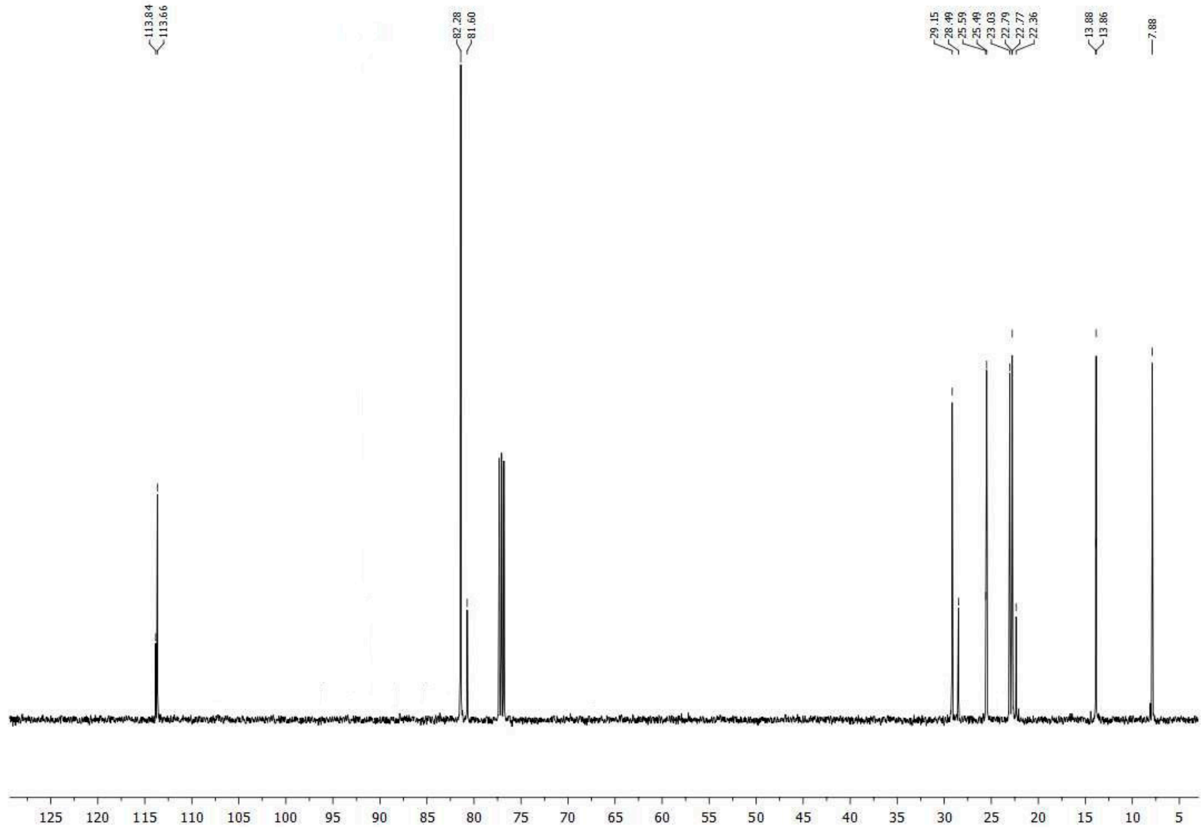


**$^{13}$C-NMR spectrum of 3-butyl-3-ethyl-1,2,4,5,7-tetraoxathiocane (12).**

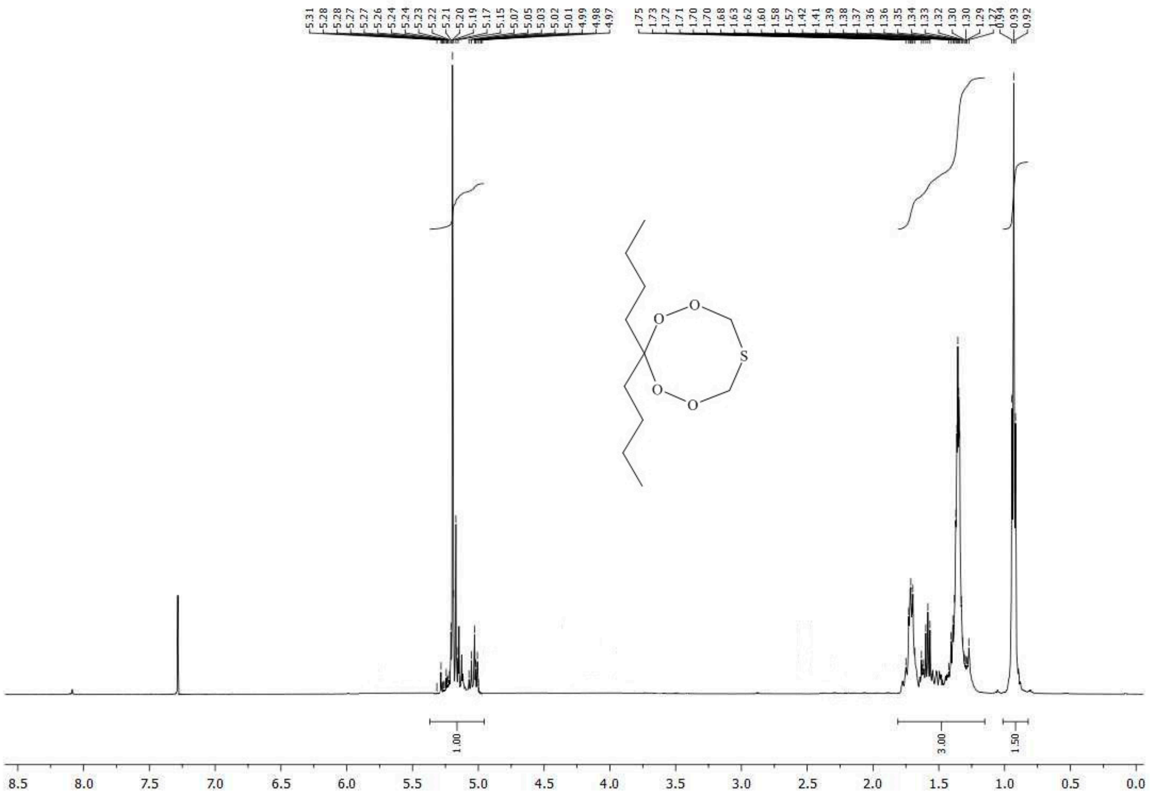

**$^{1}$H-NMR spectrum of 3,3-dibutyl-1,2,4,5,7-tetraoxathiocane (13).**

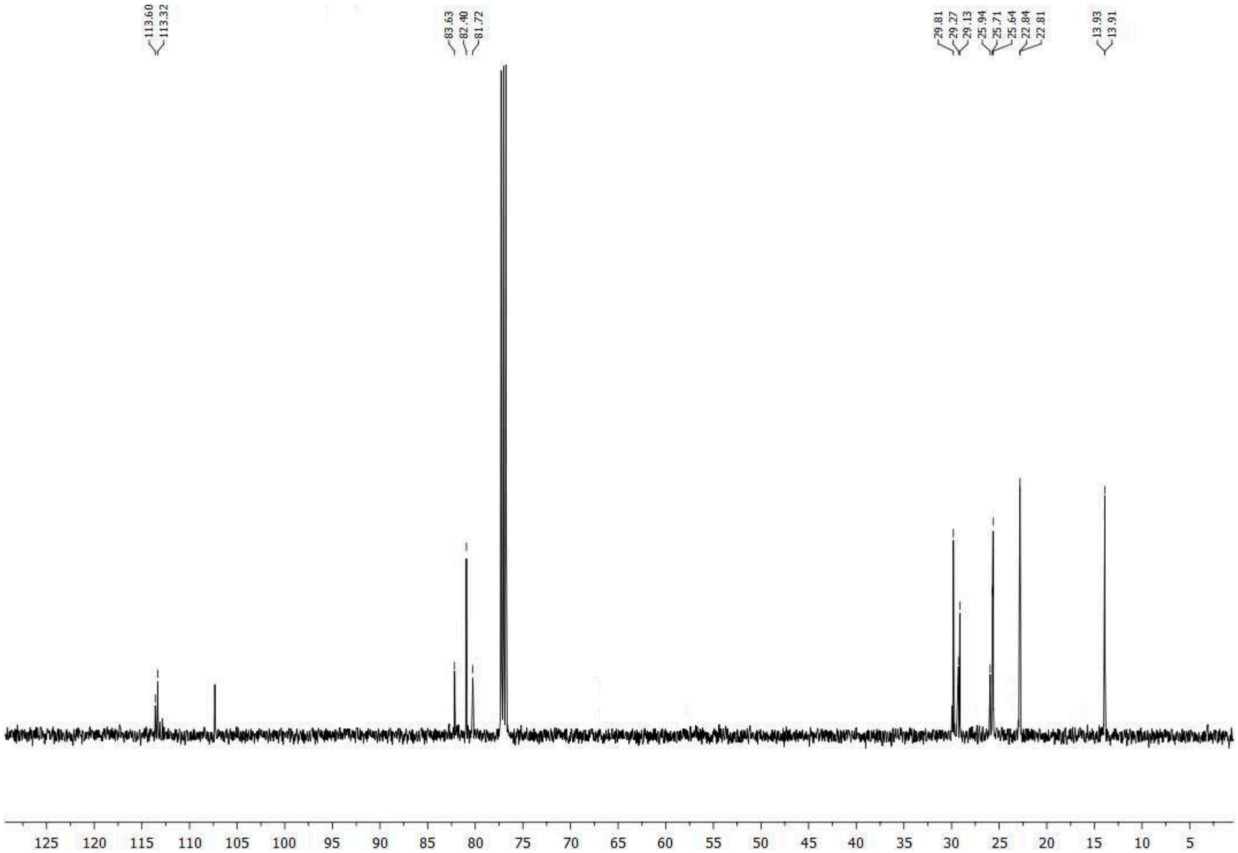

**$^{13}$C-NMR spectrum of 3,3-dibutyl-1,2,4,5,7-tetraoxathiocane (13).**

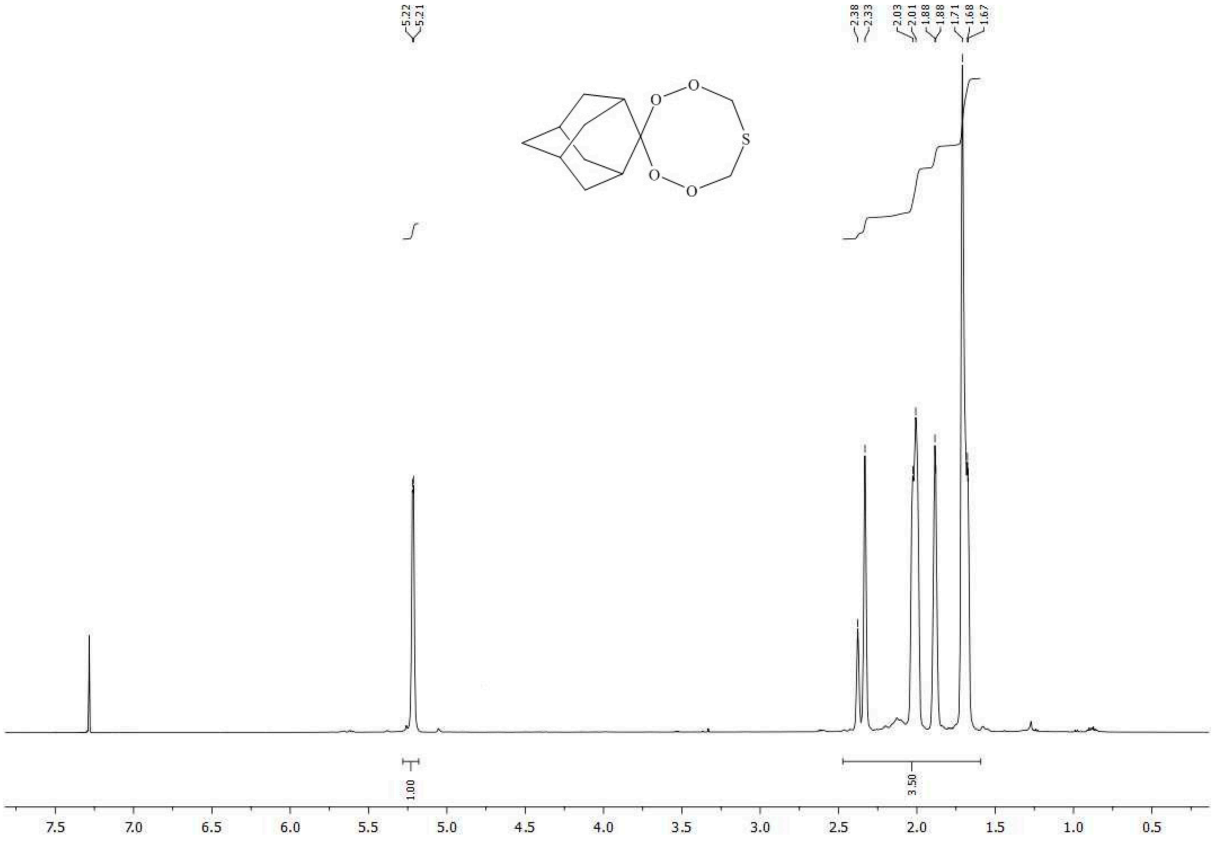


**$^{1}$H-NMR spectrum of 3-(Adamantyl-2-yl)-1,2,4,5,7-tetraaoxatioocane (14).**

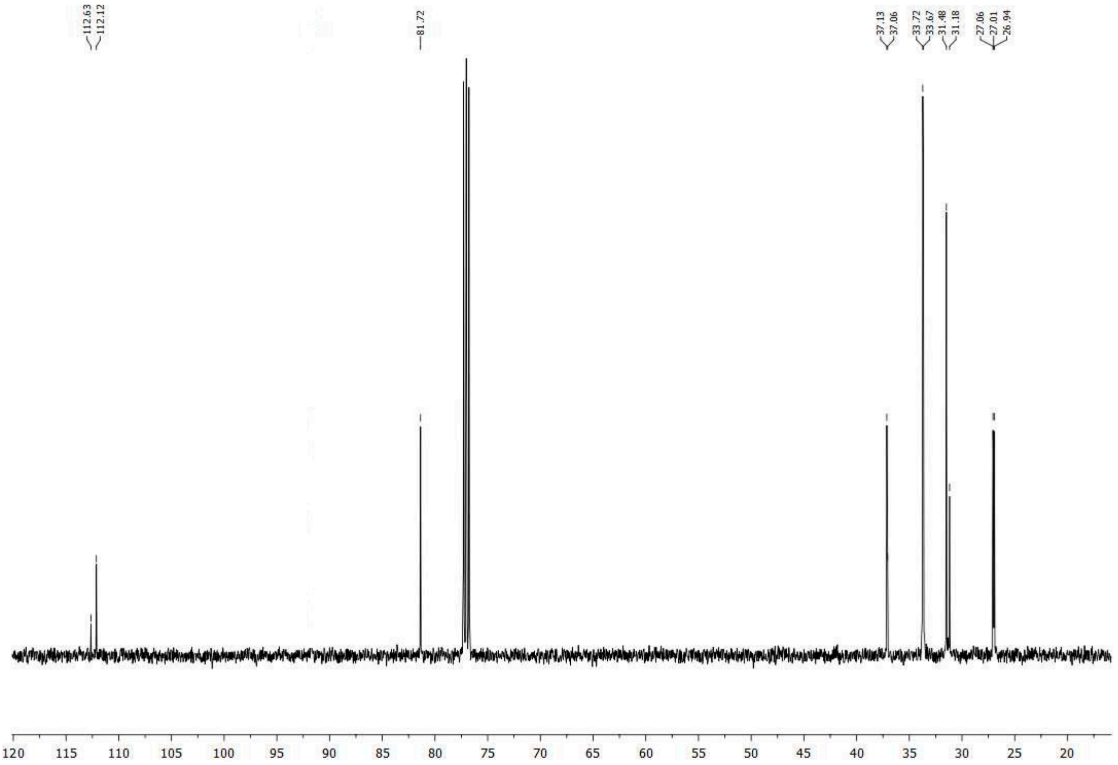


**$^{13}$C-NMR spectrum of 3-(Adamantyl-2-yl)-1,2,4,5,7-tetraoxatiocane (14).**

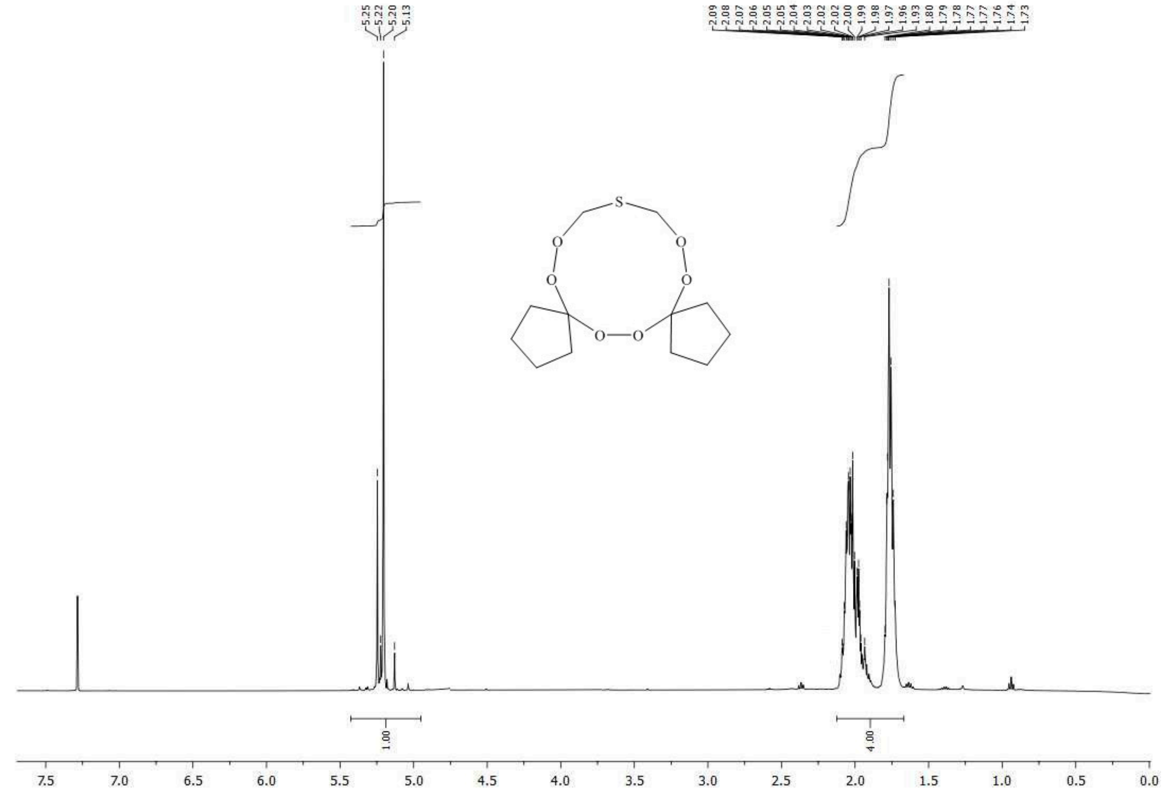


**$^{1}$H-NMR spectrum of 6,7,13,14,18,19-hexaoxa-16-thiadispiro[4.2.**$4^{8}.7^{5}$]nonadecane (**15**).

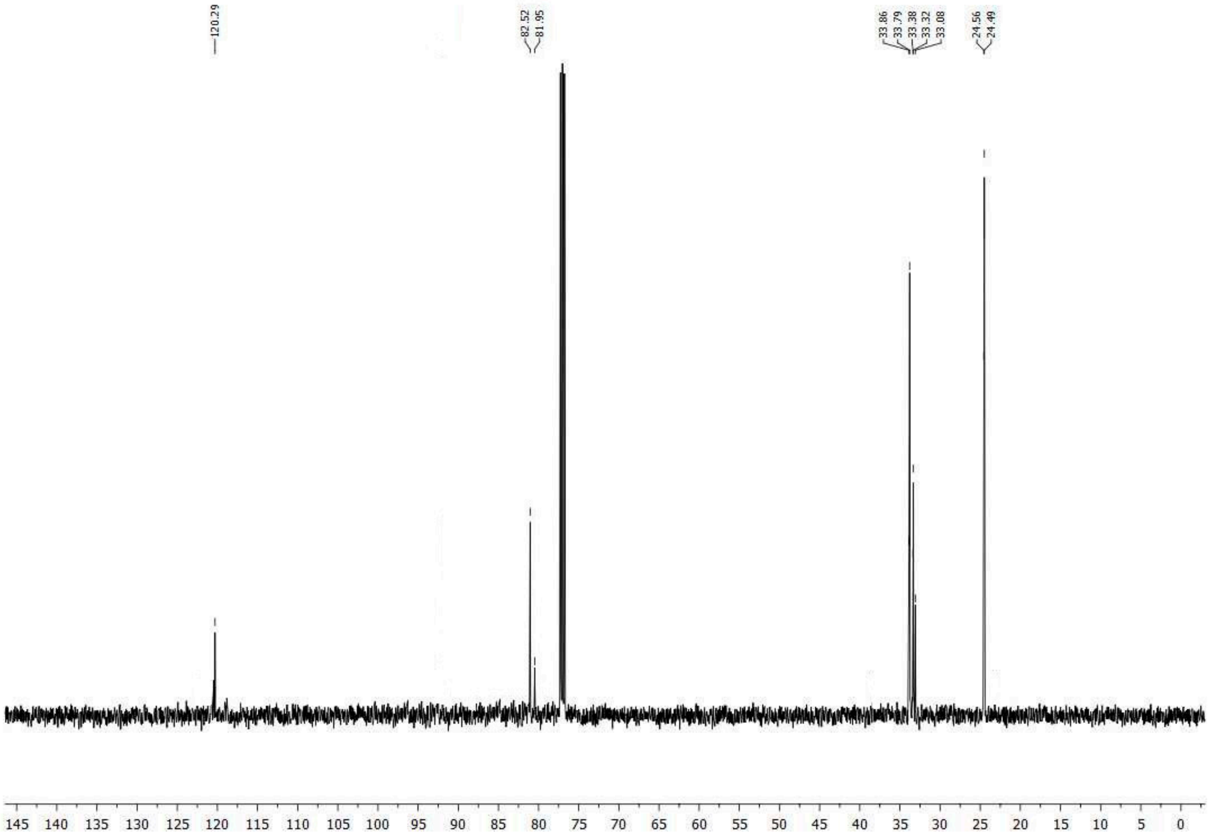


**$^{13}$C-NMR spectrum of 6,7,13,14,18,19-hexaoxa-16 (15)**.

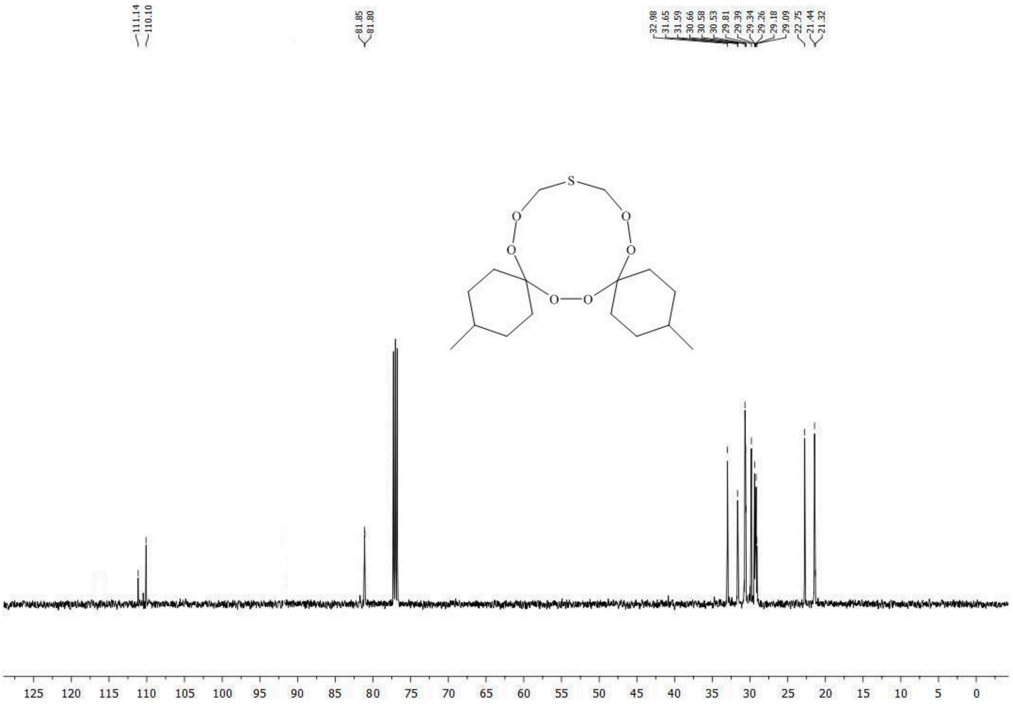


$^{1}$H-NMR spectrum of 3,12-dimethyl-7,8,15,16,20,21-hexaoxa-18-thiadispiro[5.2.5$^{9}$.7$^{6}$]henicosane (**16**).

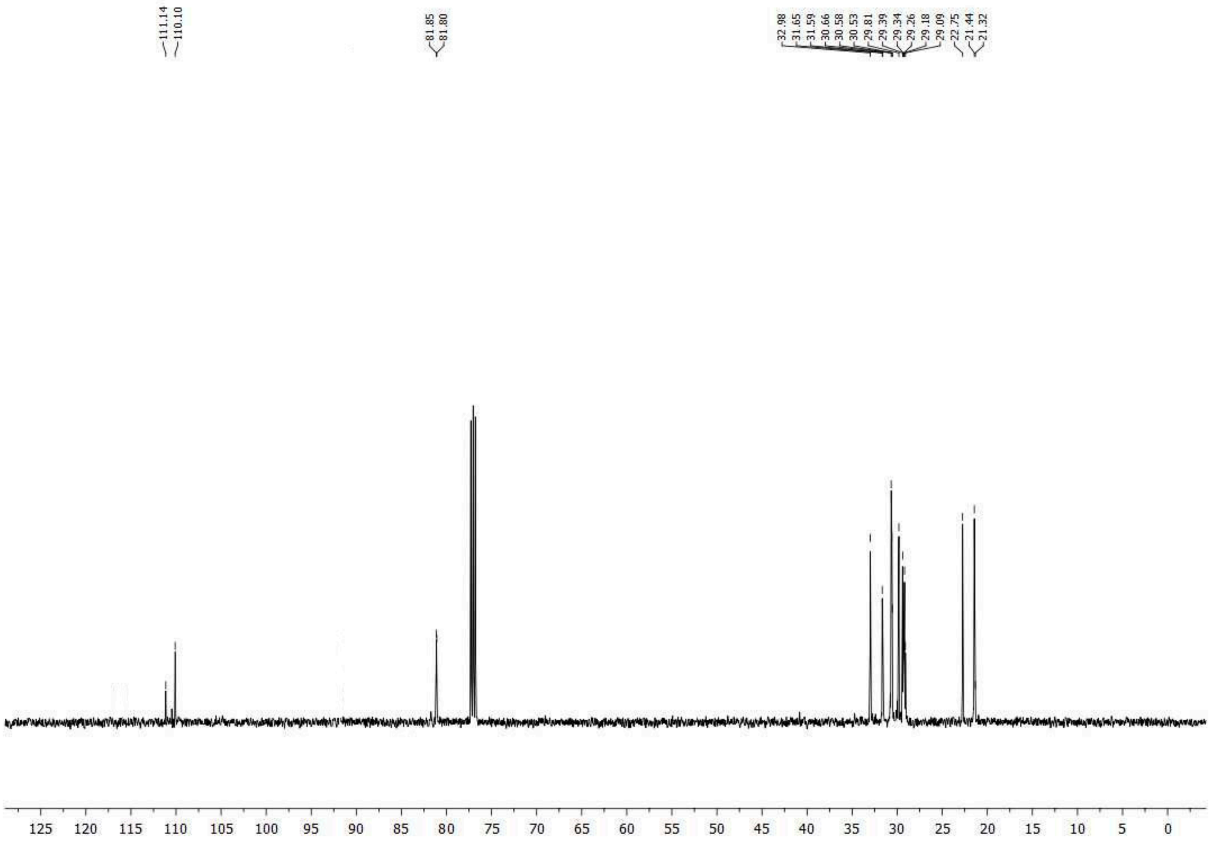


$^{13}$C-NMR spectrum of 3,12-dimethyl-7,8,15,16,20,21-hexaoxa-18-thiadispiro[5.2.5$^{9}$.7$^{6}$]henicosane (**16**).

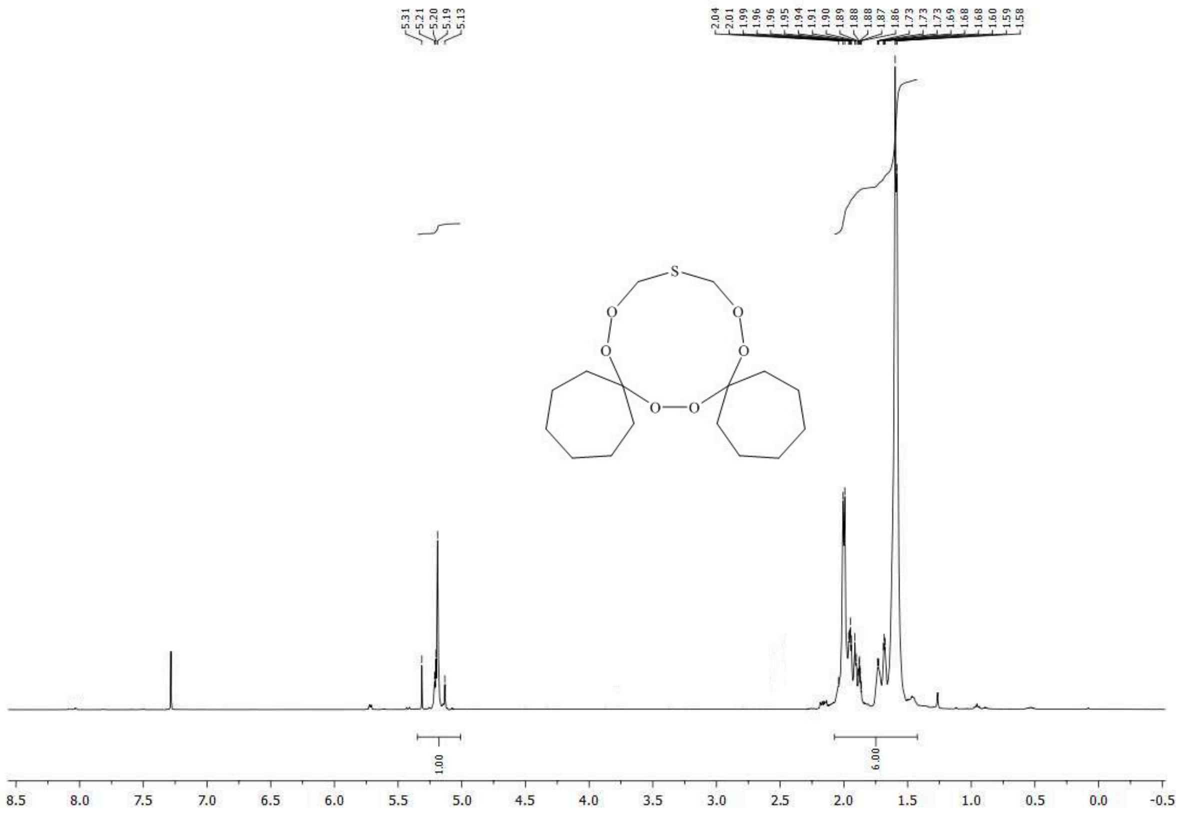


**¹H-NMR spectrum of 8,9,17,18,22,23-hexaoxa-20-thiadispiro[6.2.**$6^{10}.7^{7}$]tricosane (**17**).

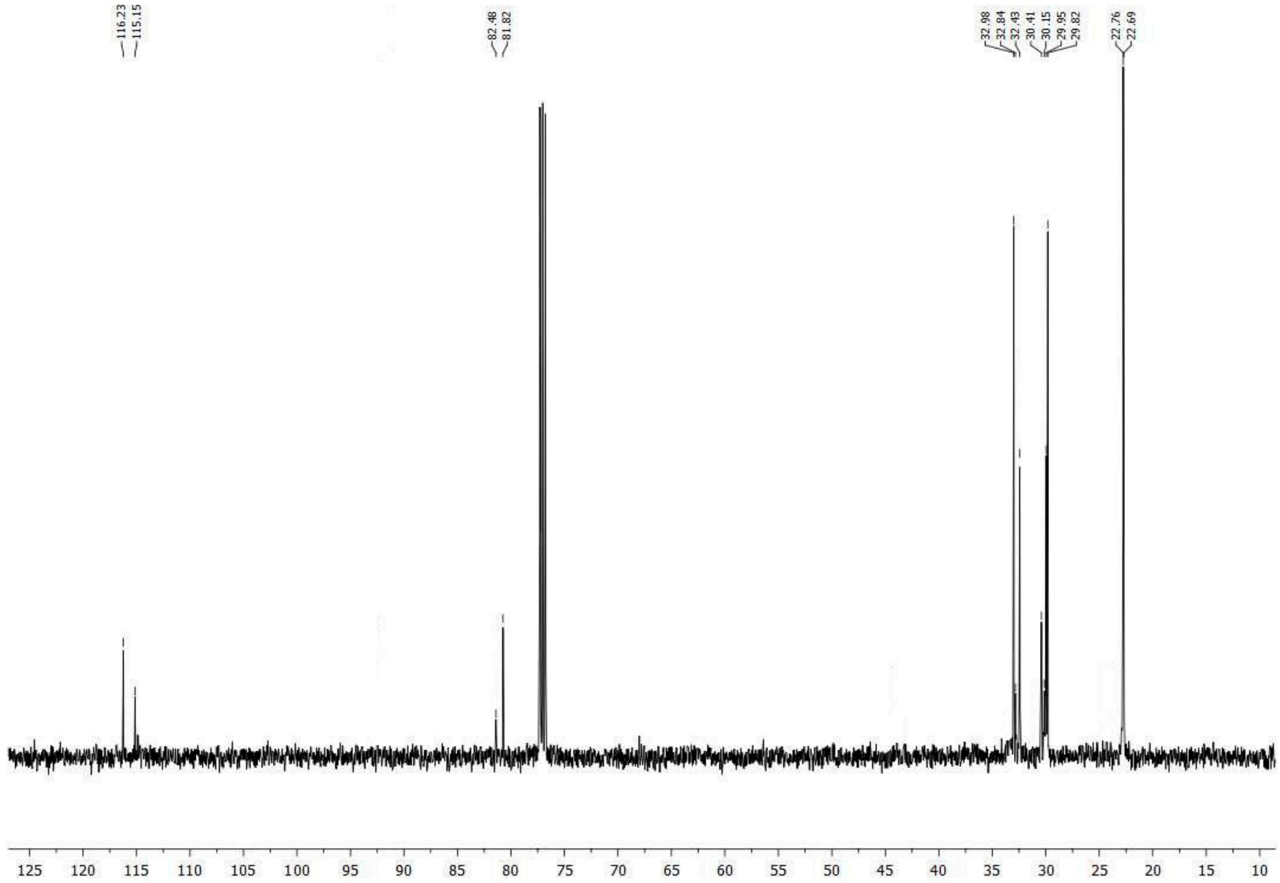


**¹³C-NMR spectrum of 8,9,17,18,22,23-hexaoxa-20-thiadispiro[6.2.**610.77]tricosane (**17**).

## 3. References

1. Makhmudiyarova, N. N.; Khatmullina, G. M.; Rakhimov, R. S.; Ibragimov, A. G.; Dzhemilev, U. M. *Arkivoc*, 2016, 427.
2. Makhmudiyarova, N. N.; Ishmukhametova, I. R.; Tyumkina, T. V.; Ibragimov A. G.; Dzhemilev, U. M. *Tetrahedron Lett*., 2018, **59**, 3161.
